# Hypothalamic CRH neurons orchestrate complex behaviours after stress

**DOI:** 10.1038/ncomms11937

**Published:** 2016-06-16

**Authors:** Tamás Füzesi, Nuria Daviu, Jaclyn I. Wamsteeker Cusulin, Robert P. Bonin, Jaideep S. Bains

**Affiliations:** 1 Department of Physiology and Pharmacology, Hotchkiss Brain Institute, Cumming School of Medicine, University of Calgary, 3330 Hospital Drive NW, Calgary, Alberta, Canada T2N 4N1; 2 Leslie Dan School of Pharmacy, University of Toronto, 144 College Street, Toronto, Ontario, Canada M5S 3M2

## Abstract

All organisms possess innate behavioural and physiological programmes that ensure survival. In order to have maximum adaptive benefit, these programmes must be sufficiently flexible to account for changes in the environment. Here we show that hypothalamic CRH neurons orchestrate an environmentally flexible repertoire of behaviours that emerge after acute stress in mice. Optical silencing of CRH neurons disrupts the organization of individual behaviours after acute stress. These behavioural patterns shift according to the environment after stress, but this environmental sensitivity is blunted by activation of PVN CRH neurons. These findings provide evidence that PVN CRH cells are part of a previously unexplored circuit that matches precise behavioural patterns to environmental context following stress. Overactivity in this network in the absence of stress may contribute to environmental ambivalence, resulting in context-inappropriate behavioural strategies.

## Abstract (web summary)

Animals exhibit a number of complex behaviours following stressful events, although the underlying circuitry is undetermined. Here, the authors use optogenetic targeting to identify a role for corticotrophin releasing hormone cells in the paraventricular nucleus in regulating such behavioural responses to acute stress.

## Graphical abstract

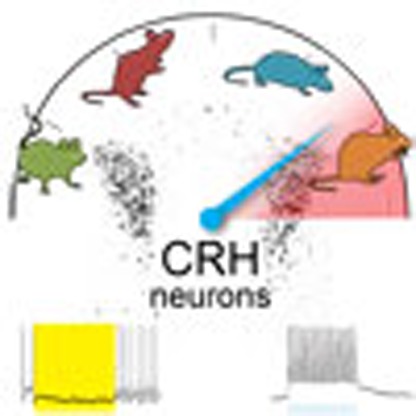

In all organisms, a perceived threat triggers specific behavioural changes and accompanying physiological responses to ensure survival^1^,^2^. The underlying circuitry responsible for these rapid defensive behaviours at the onset of stress has been studied extensively^3^,^4^. Less is known about the behaviours that follow immediately after a stressful event. Current descriptions of these behaviours indicate that they are complex and disparate; they include behaviours associated with environmental assessment, vigilance and risk avoidance (that is, locomotion, exploration, neophobia)^5^ but also behaviours that are self-directed and seemingly ambivalent to external cues (that is, self-grooming)^6^,^7^,^8^,^9^. Given the behavioural pathologies that can develop after exposure to stressful situations^10^, a clearer understanding of the underlying neural circuitry controlling behaviours after an acute stress will provide a new framework for studying the germination of stress-related disorders.

Here we used an acute, experimental stress (footshock) to launch the stress response and then studied the entire behavioural repertoire in different environmental contexts after the termination of the stress. We hypothesized that individual behaviours after stress are part of a broader behavioural pattern comprised of multiple behaviours^11^ that allows animals to return to spontaneous behaviours. We focused specifically on corticotropin-releasing hormone (CRH) cells of the paraventricular nucleus (PVN) of the hypothalamus. These cells are responsible for launching the endocrine component of the mammalian stress response^12^, but there are indications they may also regulate complex behaviours after stress. This idea is supported by reports that electrical activation of PVN and surrounding regions initiates self-grooming^7^,^8^, a behaviour observed after stress in many species in both experimental and natural conditions^8^,^9^,^13^. The canonical view of PVN CRH neurons as endocrine cells that initiate a hormonal cascade that may require tens of minutes to affect brain circuits^14^ is at odds with a role for these cells in driving rapid behaviours after stress. A more plausible scenario is that in addition to sending terminal axons to the blood vessels in the median eminence, PVN CRH neurons also send collateral projections to neighbouring hypothalamic regions^15^. These reports provide a plausible alternative through which PVN CRH neurons may control rapid behaviours after stress^16^.

Here we used cell-specific tools to directly target PVN CRH neurons^17^ and test the hypothesis that these neurons play a key role in orchestrating complex behaviours after stress. Our findings indicate that: (i) behaviours exhibit an organized structure after stress; (ii) this organization has distinct, but flexible temporal features that are sensitive to PVN CRH neuron activity and environment; (iii) There is a reciprocal relationship between environmental cues and PVN CRH neural activity in controlling specific behaviours. Understanding the specific nodes that control the behavioural sequence after stress may offer unique insights that facilitate our understanding of how the brain helps to re-set after stress.

## Results

### Quantifying multiple behaviours after stress

**Figure 1:**
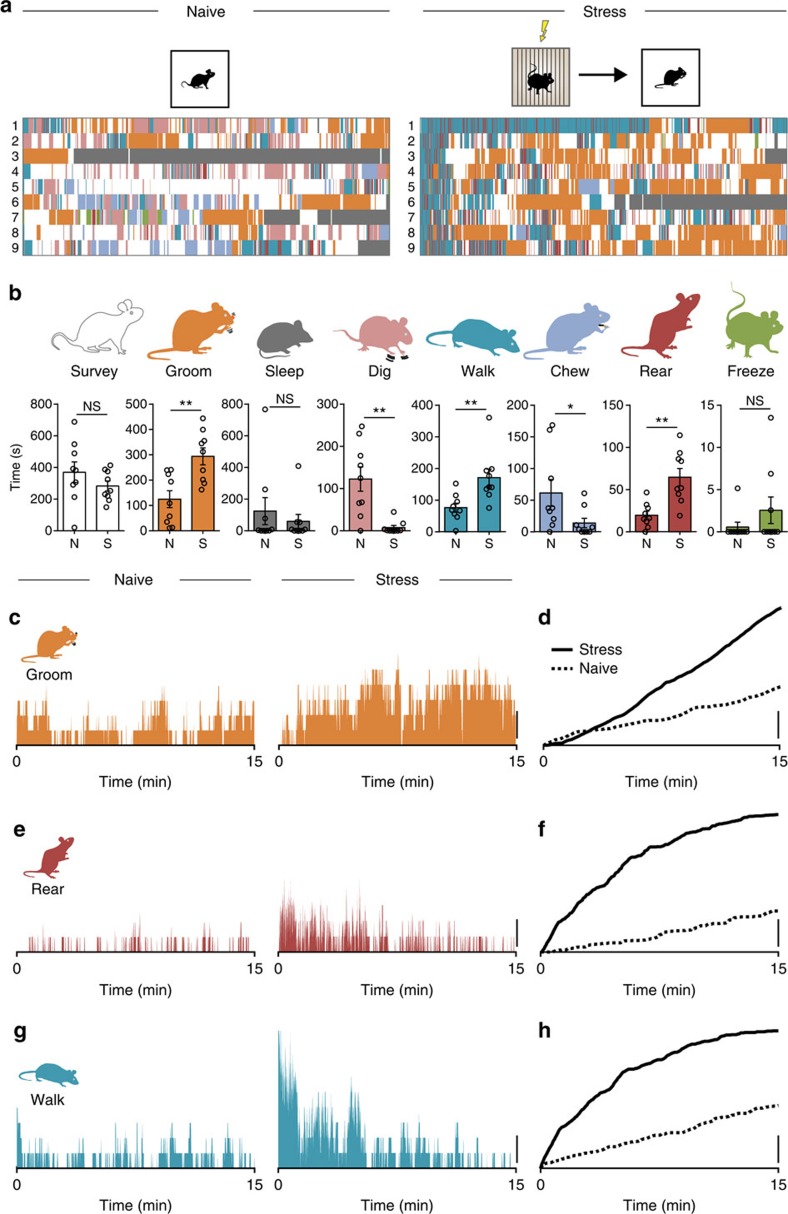
Distinct and temporally organized behavioural patterns emerge following stress. (**a**) Quantification of behavioural activity in 15-min epochs in homecage (HC) of naïve mice and mice immediately after footshock. Eight distinct behaviours are evident in naïve (N, left) and stressed (S, right) mice. Each row represents one mouse. (**b**) Grooming (naïve: 124.8±33.2 s, *n*=9 versus stressed: 294.0±33.4 s, *n*=9; *P*=0.0024; *t*-test), rearing (naïve: 19.7±4.8 s, *n*=9 versus stressed: 64.7±10.0 s, *n*=9; *P*=0.0012; *t*-test) and walking (naïve: 76.4±14.2 s, *n*=9 versus stressed: 171.4±27.0 s, *n*=9; *P*=0.0067; *t*-test) are increased after stress. Time spent digging (naïve: 122.5±28.9 s, *n*=9 versus stressed: 7.5±5.0 s, *n*=9; *P*=0.0012; *t*-test) and chewing (naïve: 61.5±21.1 s, *n*=9 versus stressed: 14.0±7.4 s, *n*=9; *P*=0.0492; *t*-test) are decreased. Surveying (naïve: 369.9±65.1 s, *n*=9 versus stressed: 282.8±31.6 s, *n*=9; *P*=0.2463; *t*-test), sleeping (naïve: 124.3±85.3 s, *n*=9 versus stressed: 59.5±44.4 s, *n*=9; *P*=0.5096; *t*-test) and freezing (naïve: 0.6±0.6 s, *n*=9 versus stressed: 2.6±1.6 s, *n*=9; *P*=0.2541; *t*-test) are unaffected. (**c**–**h**) Percentage of animals exhibiting stated behaviour at each timepoint and cumulative graphs illustrating the relative extent of grooming (**c**,**d**), rearing (**e**,**f**) and walking (**g**,**h**). Scale bars: **c**–**h**, 20%; NS, not significant; *P<0.05; **P<0.01; Error bars±s.e.m.

To examine the effects of a single episode of stress (footshock) on mouse behaviour, we first quantified all the behaviours in a 15-min observation period in the homecage (HC). We were able to discern eight distinct behaviours: surveying, grooming, digging, walking, chewing, rearing, freezing and sleeping (Fig. 1a). These behaviours appeared random with no obvious pattern or bias evident during the observation period (Fig. 1a–g). Another group of mice were transferred to a footshock chamber, subjected to a series of footshocks and then returned to the HC for observation. Although the same eight behaviours were observed, there were a number of differences: Specifically, there was a significant increase in grooming (Fig. 1b–d, Supplementary Fig. 1a,b and Supplementary Movie 1), rearing (Fig. 1b,e,f) and walking (Fig. 1b,g,h). There was a significant decrease in digging and chewing. Surveying, sleeping and freezing were unaffected. We next focused on the temporal organization specifically of the behaviours that were increased after stress. Before stress, these behaviours exhibited no discernable temporal bias or organization (Fig. 1c–h); the median time for any given behaviour was not significantly different from the halftime (HT) of the observation period (HT=450 s; Supplementary Fig. 1c–e). Furthermore, grooming, rearing and walking all showed a linear cumulative increase during the observation period. After stress, the median time for grooming did not shift from HT (Supplementary Fig. 1c). There was, however, a significant shift in the median times for rearing (Supplementary Fig. 1d) and walking (Supplementary Fig. 1e) towards the start of the observation period. These observations indicate that mice use the same behavioural palette of individual behaviours before and after stress, but the organization of these behaviours and the time allocated to each behaviour is different. Specifically, immediately after stress, there is a bias towards exploratory behaviours that wanes during the observation period and a reliable increase in grooming behaviour. This analysis provides a template for a behavioural pattern immediately after an acute stress, which can now be used to explore neural circuitry.

### Photoinhibition of PVN CRH neurons after stress

**Figure 2:**
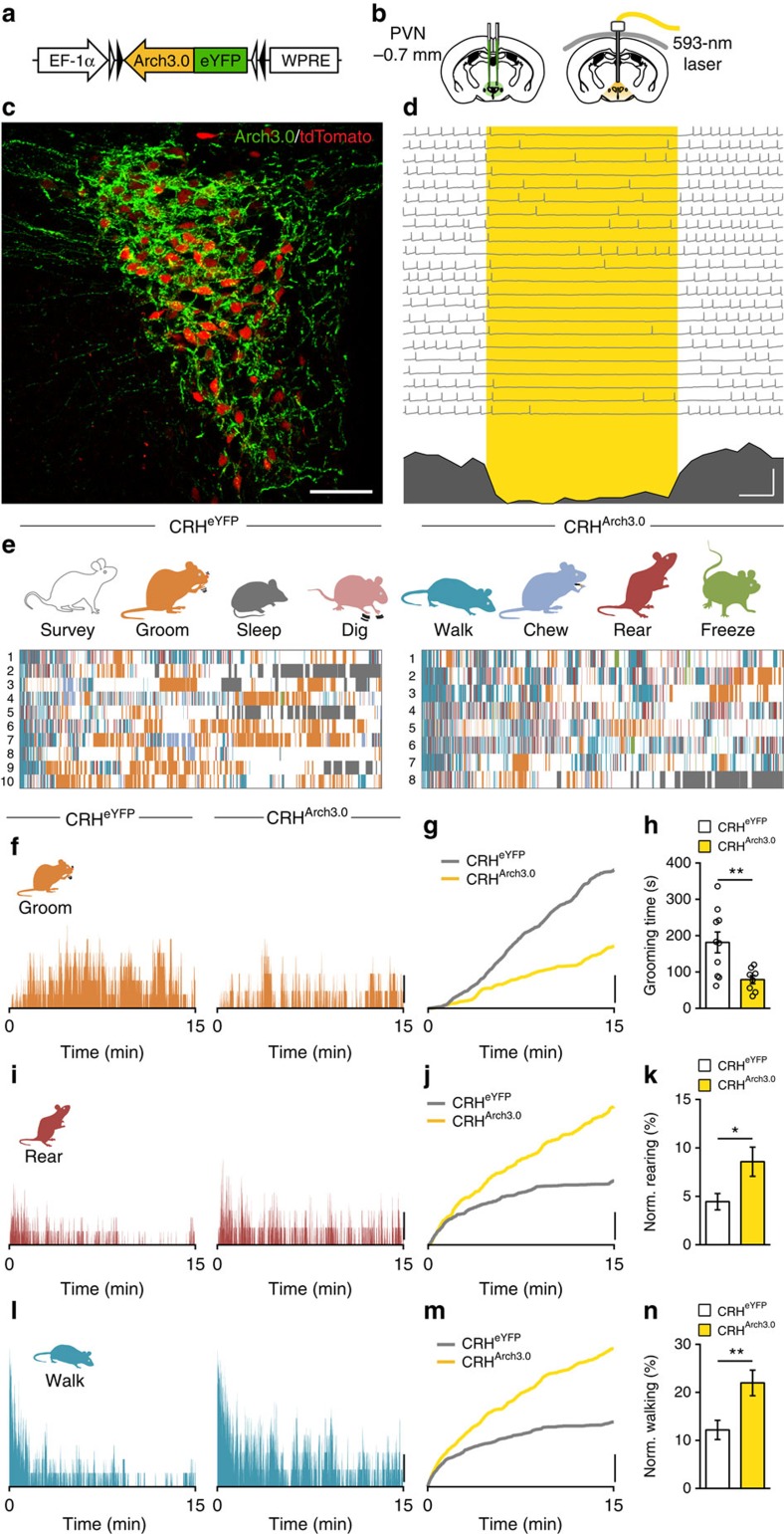
Photoinhibition of PVN CRH^Arch3.0^ neurons disrupts behavioural patterns after stress. (**a**) Cre-dependent AAV-DIO-Arch3.0-eYFP virus injected into the PVN. (**b**) Schematic maps show the injection site (left) and the implantation site of the light ferrule (right). (**c**) Confocal image shows expression of Arch3.0-eYFP (green) and tdTomato (red) in the PVN. (**d**) Delivery of yellow light to the slice (denoted by yellow box) decreases firing in PVN CRH neurons in current clamp. Bottom, Summary histogram below of action potentials form repeated trials. (**e**) Detailed analysis shows all eight behaviours in CRH^eYFP^ (left) and CRH^Arch3.0^ (right) animals in a 15-min epoch immediately after footshock. Each row represents one animal. (**f**) Histograms show percentage of animals grooming in each group during the 15-min observation period. (**g**) Cumulative graph illustrates the relative grooming in each condition. (**h**) Summary graphs show grooming is inhibited during optical inhibition of PVN CRH neurons (CRH^eYFP^: 181.4±28.7, *n*=10 versus CRH^Arch3.0^: 79.0±11.2, *n*=8; *P*=0.0080; *t*-test). (**i**) Histograms show percentage of animals rearing in each group during the 15-min observation period. (**j**) Cumulative graph illustrates the relative rearing. (**k**) Rearing time, as a fraction of all non-grooming behaviours, is increased during photoinhibition (CRH^eYFP^: 4.5±0.8%, *n*=10 versus CRH^Arch3.0^: 8.6±1.5%, *n*=8; *P*=0.0229; *t*-test). (**l**) Histograms show percentage walking in each group during the 15-min observation period. (**m**) Cumulative graph illustrates the relative walking. (**n**) Fractional walking time is also increased during photoinhibition of PVN CRH neurons (CRH^eYFP^: 12.2±2.0%, *n*=10 versus CRH^Arch3.0^: 22.0±2.7%, *n*=8; *P*=0.0084; *t*-test). Scale bars: (**c**), 50 μm; (**d**), 2 Hz and 1 s; (**f**,**g**,**i**,**j**,**l**,**m**), 20%; NS, not significant, *P<0.05; **P<0.01; Error bars±s.e.m.

The robust increase in grooming after stress has been reported previously^7^,^18^ and it has been suggested that PVN neurons may control the precise timing of grooming with respect to other behaviours^19^. Other studies, however, report that PVN lesions fail to affect grooming following stress^20^. To directly assess the contribution of PVN CRH neurons in grooming and related behaviours after stress, we used mice expressing Archaerhodopsin 3.0 in CRH neurons (CRH^Arch3.0^)^21^ to attenuate firing specifically in PVN CRH neurons (Fig. 2a–d). The effects of light on behaviour were always compared with control CRH^eYFP^ mice^22^,^23^ (Supplementary Movie 1). First, using brain slices, we confirmed that delivery of yellow light reliably inhibited firing in CRH neurons from CRH^Arch3.0^ mice (frequency of action potentials: 7.7±6.7% of baseline *P*=0.0168, *n*=5, repeated measures one-way analysis of variance (ANOVA)). In addition, we did not observe any rebound increases in activity of CRH neurons following termination of optical inhibition (frequency of action potentials: 62.5±51.0% of baseline, *P*>0.9999, *n*=5, repeated measures one-way ANOVA). Next, mice were exposed to footshock, and continuous yellow light was delivered into the PVN during HC observation for 15 min (Fig. 2e, Supplementary Fig. 2a,b and Supplementary Movie 2). This decreased the time spent grooming (Fig. 2f–h and Supplementary Fig. 2c,d), increased time spent rearing (Fig. 2i–k and Supplementary Fig. 2e) and walking (Fig. 2l–n and Supplementary Fig. 2f). The temporal organization of these behaviours, however, was unaltered (Supplementary Fig. 3l–n). Other behaviours were unaffected by photoinhibition of CRH neurons (Supplementary Fig. 2g–k). To determine whether the relative changes in total time spent on a given behaviour reflected a simple re-allocation of time or whether specific behaviours were favoured after silencing CRH neurons, we examined the relative time spent rearing or walking after removing the time allocated to grooming. There was a significant increase in fractional time rearing (Fig. 2k) and walking (Fig. 2n) indicating that these behaviours are preferentially increased after silencing PVN CRH neurons. Since stress increases circulating glucocorticoids (CORT)^12^, we probed the potential link between CORT and grooming. Blocking CORT synthesis 1 h before footshock blunted CORT increases in response to footshock (Supplementary Fig. 3a,b), but had no effect on grooming (Supplementary Fig. 3c) or rearing (Supplementary Fig. 3d). To assess the role of PVN CRH neurons in the regulation of behaviour in the absence of stress, we silenced the CRH neurons of naïve mice. We observed no difference in the behavioural pattern of the mice in response to optical inhibition in naïve condition (Supplementary Fig. 4a–d). Taken together, these observations indicate that persistent activity of PVN CRH neurons following footshock is necessary for regulating specific behaviours, but this occurs independently of CORT.

### Photoactivation of PVN CRH neurons

**Figure 3:**
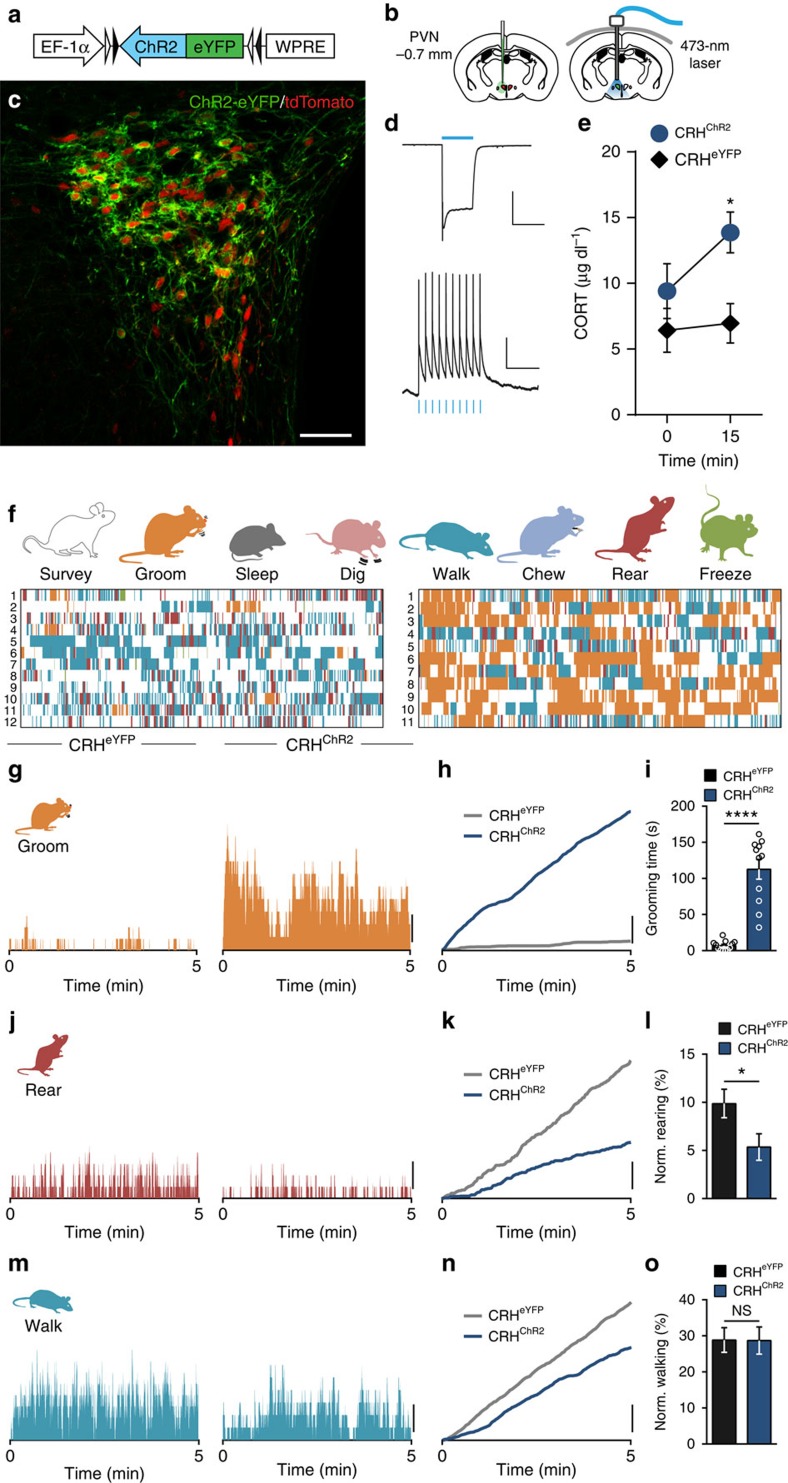
Photostimulation of PVN CRH^ChR2^ neurons triggers behaviours in the absence of stress. (**a**) Construct of Cre-dependent AAV-DIO-ChR2-eYFP virus. (**b**) Schematic maps show the injection of virus into the PVN of CRH-Cre/tdTomato mice (left) and the implantation site of the light ferrule (right). (**c**) Confocal image shows expression of ChR2-eYFP (green) and tdTomato (red) in the PVN. (**d**) Optical stimulation in current clamp (top) and voltage clamp (bottom) shows delivery of blue light reliably controls PVN CRH neurons. (**e**) Blood samples taken before and 15 min after the onset of optical stimulation show increase in CORT levels specifically in CRH^ChR2^ mice (CRH^eYFP^: 0.528 μg dl^−1^ increase, *n*=6; versus CRH^ChR2^: 5.327 μg dl^−1^ increase, *n*=5; *P*=0.0316; *t*-test). (**f**) Detailed analysis shows the pattern of eight different behaviours observed in CRH^eYFP^ (left) and CRH^ChR2^ (right) animals during 5 min of optical stimulation in an observational chamber to which mice were previously habituated to in the absence of stress. Each row represents one animal. (**g**) Histograms show percentage of animals grooming in each group during optical stimulation. (**h**) Cumulative graph illustrates relative extent grooming. (**i**) Optical stimulation of PVN CRH neurons increased grooming time (CRH^eYFP^: 6.8±1.9 s, *n*=12; versus CRH^ChR2^: 112.6±13.6 s, *n*=11; *P*<0.0001; *t*-test). (**j**) Histograms show percentage of animals rearing in each group during optical stimulation. (**k**) Cumulative graph illustrates relative rearing. (**l**) Rearing time as a fraction of non-grooming behaviours is decreased by photostimulation of CRH neurons (CRH^eYFP^: 9.9±1.5%, *n*=12; versus CRH^ChR2^: 5.4±1.4%, *n*=11; *P*=0.0396; *t*-test). (**m**) Histograms show percentage of animals walking during the optical stimulation. (**n**) Cumulative graph illustrates relative walking. (**o**) Fractional walking time is unaltered by optical stimulation (CRH^eYFP^: 28.8±3.4%, *n*=12; versus CRH^ChR2^: 28.7±3.8%, *n*=11; *P*=0.9784; *t*-test). Scale bars: **c**, 50 μm; **d**, Top: 200 pA and 500 ms, Bottom: 20 mV and 200 ms; (**g**,**h**,**j**,**k**,**m**,**n**), 20%; NS, not significant, *P<0.05; ****P<0.0001; Error bars±s.e.m.

To probe the effects of CRH neuron activation, in the absence of an external stress, we expressed Channelrhodopsin 2 (ChR2) unilaterally in CRH neurons (CRH^ChR2^)^21^ (Fig. 3a-c). In brain slices, we confirmed that blue light induces inward currents and spiking in CRH^ChR2^ neurons reliably at frequencies up to 20 Hz (Fig. 3d). To control CRH activity *in vivo,* we implanted a fibre optic probe ipsilateral to the injection site (Supplementary Fig. 5a,b). As expected, photostimulation of the PVN in CRH^ChR2^ mice increased circulating CORT (Fig. 3e) and increased the number of c-Fos-positive cells in the PVN (Supplementary Fig. 5c–e). Next, we photostimulated naïve (unstressed) CRH^ChR2^ mice in an observational chamber to which mice had been habituated (HAB). This elicited robust grooming (Supplementary Fig. 6a and Supplementary Movie 3), with a rapid onset. Behaviour ceased immediately when photostimulation was terminated (Supplementary Fig. 6a). To examine the effects of PVN CRH activation in the HAB environment and to assess different behaviours, we conducted additional experiments during which blue light was delivered for 5  min (Fig. 3f). Photostimulation increased grooming (Fig. 3g–i) throughout the 5-min observation period. It also consistently decreased both absolute rearing (Fig. 3j–l and Supplementary Fig. 6b) and fractional rearing (Fig. 3l). There was no effect of photostimulation on walking (Fig. 3m–o and Supplementary Fig. 6c) or surveying (Supplementary Fig. 6d,e). We next asked whether these changes in behaviour were sensitive to changing the frequency of photostimulation. We observed a linear increase in grooming with increasing frequencies from 1 to 20 Hz. This was accompanied by a progressive, frequency-dependent decrease in rearing (Supplementary Fig. 6f). Optical stimulation of PVN CRH neurons did not affect the temporal organization of either grooming, rearing or walking (Supplementary Fig. 6g-i). These observations demonstrate that specific activation of PVN CRH is sufficient to increase grooming and decrease rearing.

### PVN CRH neurons target a discrete cell population in LH

**Figure 4:**
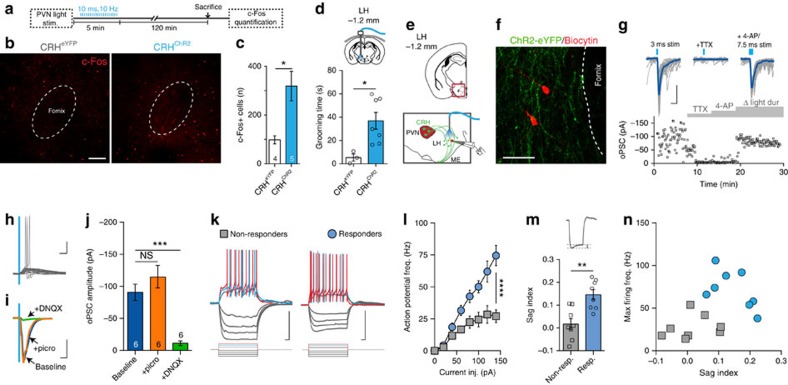
PVN CRH neurons project to the lateral hypothalamus. (**a**) Schematic of experimental design. (**b**) c-Fos-positive cells in LH of CRH^eYFP^ and CRH^ChR2^ following photostimulation in PVN. (**c**) Summary data of c-Fos in LH (CRH^eYFP^: 100.5±21.1, *n*=4 versus CRH^ChR2^: 318.8±60.4, *n*=5; *P*=0.0179; *t*-test). (**d**) *In vivo* photostimulation in LH (20hz, 5 min) increases grooming time (CRH^eYFP^: 5.4±3.0 s, *n*=3; versus CRH^ChR2^: 36.9±7.3 s, *n*=7; *P*=0.0264; *t*-test). (**e**) Schematic map and experimental design of *in vitro* whole-cell recordings from LH neurons. (**f**) Biocytin filled recorded neurons in LH (red) surrounded by ChR2-eYFP-expressing fibres (green). (**g**) In voltage clamp (HP=−80 mV), blue light (2–5 ms) elicits fast inward currents (latency: 4.7±0.3 ms) that are abolished by TTX (baseline: 103.1±2.0 pA versus TTX: 4.7±0.7 pA, *n*=8; *P*<0.0001; repeated-measures one-way ANOVA) and partially restored by increasing light-pulse duration (7.5–10 ms) during application of 4-aminopyridine (4-AP)^28^,^44^ (40.1±9.0 pA; *P*<0.0001 versus baseline; *P*=0.0222 versus TTX, *n*=8; repeated-measures one-way ANOVA). (**h**) Sample traces show effects of optical stimulation on LH neuron firing. (**i**,**j**) oPSCs are unaffected by picrotoxin but are potently inhibited by DNQX (baseline: 90.7±12.6 pA, picro: 115.1±17.6 pA, DNQX: 11.3±3.5 pA, *n*=6; baseline versus DNQX, *P*=0.0007; repeated-measures one-way ANOVA). (**k**) Current clamp recordings of LH neurons reveal two distinct electrophysiological profiles. Cells depicted by grey square show no synaptic responses to blue light pulses; blue circle indicates cells with synaptic responses. (**l**) Action potential frequency–current relationship in responding/non-responding cells (*n*=16; *P*<0.0001; two-way ANOVA). (**m**) Differential hyperpolarization-induced ‘sag' between groups (non-responder: 0.017±0.024, *n*=8; responder: 0.146±0.022, *n*=8; *P*=0.0016; *t*-test). Sag index calculation: (Vm max—Vm steady state)/Vm max, in response to −80 pA hyperpolarizing step). (**n**) Firing frequency (+60 pA step) versus sag index in responding and non-responding cells. Scale bars: (**b**) and (**f**), 50 μm; (**g**) and (**i**), 50 pA and 10 ms; (**h**) and (**k**), 50 mV and 50 ms; NS, not significant; *P<0.05; **P<0.01; ***P<0.0005; ****P<0.0001; Error bars,±s.e.m.

Axon collaterals from PVN CRH neurons have been described in the lateral hypothalamus (LH)^15^. In addition, PVN CRH neurons also express mRNA for vesicular glutamate transporter 2 (VGluT2)^24^ providing a substrate for fast synaptic transmission. To investigate the putative neural circuit downstream of CRH neurons, we delivered blue light *in vivo* for 5 min (Fig. 4a), and 2 h later, killed mice and processed brain tissue for c-Fos. There was an increase in c-Fos-positive cells in the LH (Fig. 4b,c). To directly test the contribution of the projection to LH, a fibre was unilaterally positioned in the LH to stimulate axon terminals (Fig. 4d and Supplementary Fig. 7a,b). Photostimulation of the fibres increased grooming (Fig. 4d). A network of enhanced yellow fluorescent protein (eYFP)-positive fibres with bouton-shaped structures was evident in the LH (Fig. 4e,f and Supplementary Fig. 8a–c). There were no eYFP-positive fibres in extra-hypothalamic regions known to receive input from the PVN^25^,^26^ or implicated in grooming behaviours^27^ (Supplementary Fig. 8d–i). To ask whether PVN CRH neurons projecting to LH are distinct from PVN CRH neurons that project to the median eminence, we performed a dual retrograde tracer experiment. We injected fluorogold in the tail vein (Supplementary Fig. 9a) to label neurons with axon projections that terminate outside the blood–brain barrier (endocrine) and also injected fluorescent beads into the perifornical region of the LH (Supplementary Fig. 9b) to label cell bodies that have release sites in the LH. Fluorogold and retrobeads were co-localized in a subset of PVN CRH neurons consistent with the hypothesis that individual neurons simultaneously project to the median eminence and the LH (Supplementary Fig. 9c). To directly characterize functional synaptic transmission from CRH axon fibres to LH neurons, we obtained *in vitro* whole-cell recordings from LH neurons (Fig. 4f). Based on the fast latency, blockade by tetrodotoxin, and subsequent partial recovery in the presence of 4-aminopyridine^28^, we conclude these connections are monosynaptic. Pharmacological experiments demonstrating complete block with the AMPA/kainate receptor antagonist, 6,7-dinitroquinoxaline-2,3-dione (DNQX), indicates the synapses are glutamatergic (Fig. 4g-j). In addition, we tested the effects of the CRHR1 antagonist on evoked transmission and failed to see any effect on individual excitatory postsynaptic currents or trains of excitatory postsynaptic currents (data not shown). Interestingly, not all LH neurons tested responded to photostimulation. We noted two distinct subtypes on the basis of electrical fingerprints. Cells with a pronounced delay to first spike in response to a depolarizing current injection and a linear current–voltage relationship, failed to respond to optical stimulation of CRH fibres (non-responders, Fig. 4k). By contrast, cells with a higher firing rate (Fig. 4l) and a prominent ‘sag' in the membrane potential (Fig. 4k–n) always responded to photostimulation (responders). These observations demonstrate that PVN CRH neurons send excitatory, glutamatergic projections to an electrophysiologically distinct population of neurons in the LH.

### Behavioural profiles after stress are context sensitive

**Figure 5:**
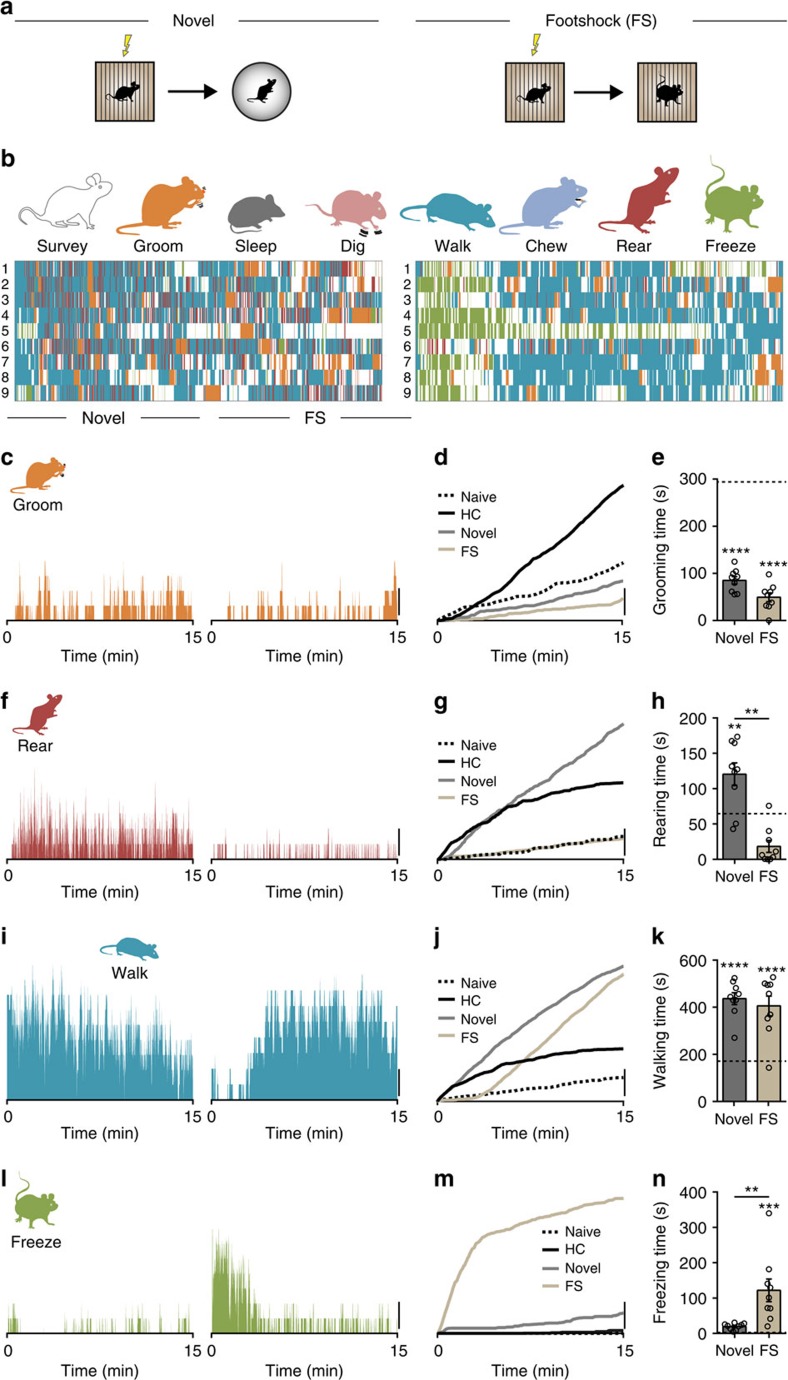
Stress-induced behavioural patterns are sensitive to context. (**a**) Schematic of experiment showing the two different environments, the novel context (Novel) and footshock chamber (FS) immediately after footshock. (**b**) Detailed analysis shows the pattern of behaviours exhibited by the animals in different contexts. Each row represents one animal. The different environments change the behavioural pattern expressed by the animal. (**c**) Percentage of animals grooming at each timepoint. (**d**) Cumulative graphs illustrate the relative grooming in different contexts including homecage (HC) immediately after stress and compared with naïve mice (Fig. 1). (**e**) Grooming is the dominant in HC (dotted line represents mean grooming time in HC; Novel: 85.1±8.0 s; FS: 49.3±9.4 s; Novel versus HC *P*<0.0001; FS versus HC *P*<0.0001; *n*=9 in each group; one-way ANOVA). (**f**) Percentage of animals rearing at each timepoint. (**g**) Cumulative graphs illustrate the relative amount of rearing in different contexts including HC immediately after stress and the naïve mice (Fig. 1). (**h**) Mice spend more time rearing in Novel (dotted line represents mean rearing time in HC; Novel: 120.4±16.0 s; FS: 18.3±8.5 s; Novel versus HC *P*=0.0097; Novel versus FS *P*<0.0001; *n*=9 in each group; one-way ANOVA). (**i**) Percentage of animals walking at each timepoint. (**j**) Cumulative graphs illustrate the relative walking time in different contexts including HC immediately after stress and in naïve mice (Fig. 1). (**k**) Mice spend the same amount of time walking in Novel and FS (dotted line represents mean walking time in HC; Novel: 436.7±25.2 s; FS: 405.6±41.7 s; Novel versus HC *P*<0.0001; FS versus HC *P*<0.0001; *n*=9 in each group; one-way ANOVA). (**l**) Percentage of animals freezing at each timepoint. (**m**) Cumulative graphs illustrate the relative freezing in different contexts including HC immediately after stress and the naïve mice (Fig. 1). (**n**) Freezing behaviour was only significant in FS (dotted line represents mean freezing time in HC; Novel: 19.5±3.0 s; FS: 121.8±32.0 s; Novel versus FS *P*=0.0021; FS versus HC *P*=0.0004; *n*=9 in each group; one-way ANOVA). Scale bars: (**c**,**d**,**f**,**g**,**i**,**j**,**l**,**m**), 20%; **P<0.01; ***P<0.0005; ****P<0.0001; Error bars±s.e.m.

The organization of behaviours into an organized repertoire after stress suggests a hardwired innate strategy. To be optimal, however, this strategy should be sensitive to changes in the animal's environment. To test this idea, we conducted experiments in which mice received a footshock and then were placed either in a novel environment (Novel) or observed in the footshock chamber (Fig. 5a). The behavioural data were compared with the animals placed into their HC after footshock (Fig. 1). Again, all eight behaviours were evident in the novel environment (Fig. 5b), but when compared with the HC, there was a decrease in grooming (Fig. 5c–e, Supplementary Fig. 10a,b and Supplementary Movie 4) and increases in both rearing (Fig. 5f–h) and walking (Fig. 5i–k). We next assessed the temporal features of these behaviours. Although there was no difference in the distribution of grooming in comparison to HC, (Supplementary Fig. 10d) rearing and walking were sustained for a longer time (Supplementary Fig. 10e,f) throughout the observation period. Mice maintained in the footshock cage (FS) showed robust freezing immediately after footshock (Fig. 5l–n, Supplementary Fig. 10g and Supplementary Movie 5). As this behaviour gradually dissipated, there was an increase in walking (Fig. 5i–k and Supplementary Fig. 10f). There was less rearing (Fig. 5f–h) and grooming (Fig. 5c–e) in the footshock chamber. These observations demonstrate that the environment has a profound effect on the behavioural pattern after stress and hints at underlying differences in the strategy adopted by the animal in matching its behavioural palette to the context.

### Context affects behaviours driven by PVN CRH photoactivation

**Figure 6:**
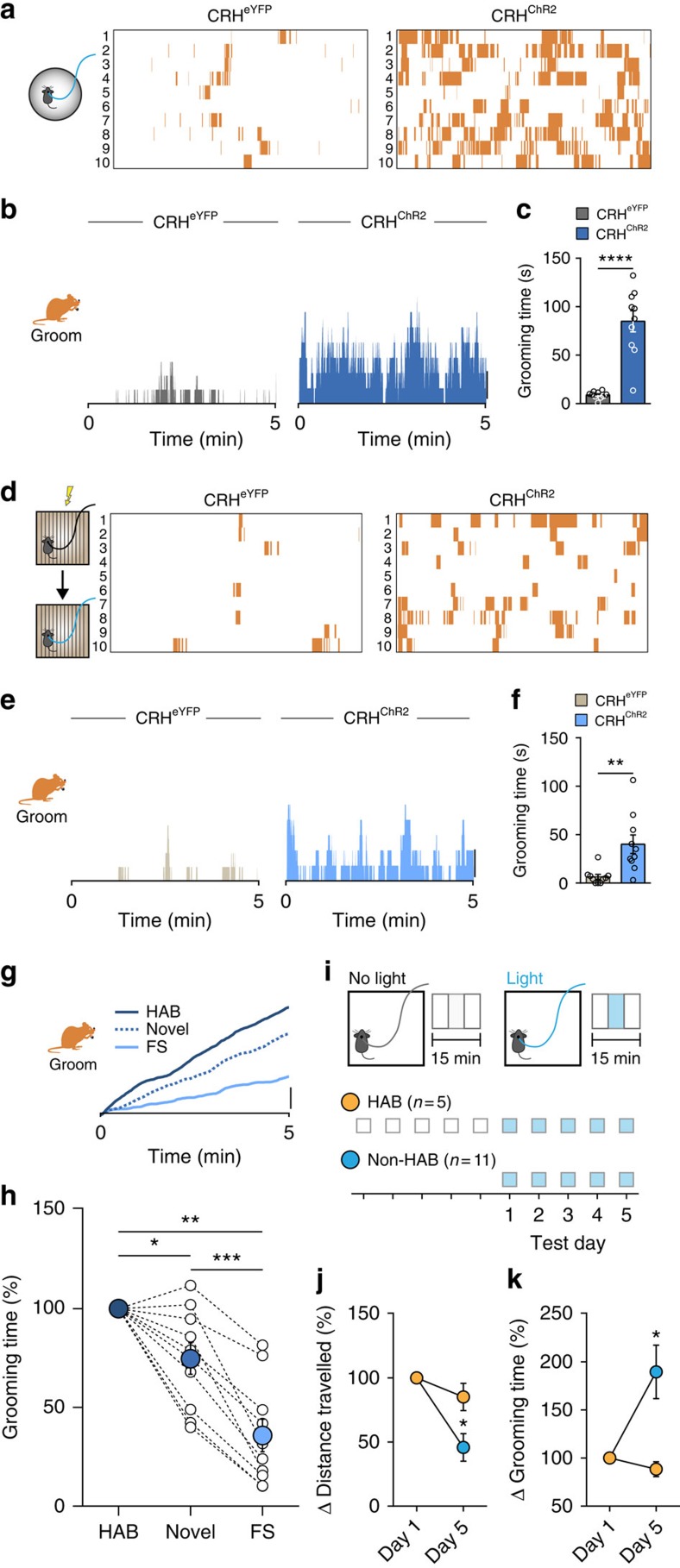
Photostimulation-induced grooming is sensitive to the context. (**a**–**h**) Identical light delivery protocol (10 hz for 5 min) used in novel environment (Novel) and in the FS immediately after footshock stress. (**a**–**c**) Photostimulation of PVN CRH neurons in Novel. (**a**) Each row represents an individual animal. (**b**) Histogram showing percentage of animals grooming. (**c**) Quantification of grooming in Novel (CRH^eYFP^: 8.9±1.2 s, *n*=10; versus CRH^ChR2^: 85.0±10.9 s, *n*=10; *P*<0.0001; *t*-test). (**d**–**f**) Optical stimulation of PVN CRH neurons in FS. (**d**) Each row represents an individual animal. (**e**) Histogram showing percentage of animals grooming. (**f**) Quantification of grooming in FS (CRH^eYFP^: 6.4±2.5 s, *n*=10; versus CRH^ChR2^: 40.1±9.5 s, *n*=10; *P*=0.0031; *t*-test). (**g**) Cumulative graphs illustrate the relative effect of different contexts on optically evoked grooming including habituated (HAB) context (data shown in Fig. 3). (**h**) Optically evoked grooming time is gradually attenuated as the presumptive threat level of the context increases (Novel: 74.7±7.9% of HAB, *P*=0.0405 versus HAB; FS: 35.8±8.3% of HAB, *P*=0.0013 versus HAB, *P*=0.0006 versus Novel; *n*=10; repeated-measures one-way ANOVA). (**i**) Schematic of experiment showing effects of habituation on ChR2-induced grooming. (**j**) Increased familiarity in the paradigm causes a decrease in baseline locomotion distance in the arena in the non-HAB (day 5: 45.79±10.78% of day 1, *n*=11; *P*=0.0004 versus day 1; paired *t*-test), but not in the HAB animals (day 5: 85.13±10.62% of day 1, *n*=5; *P*=0.21 versus day 1, paired *t*-test; *P*=0.0431 versus non-HAB, *t*-test). (**k**) Optically evoked grooming time is higher on the fifth day in non-HAB mice (day 5: 189.6±27.8% compared with the day 1, *n*=11; *P*=0.0016; paired *t*-test). In contrast, HAB animals show invariant response to optical activation (day 5: 88.5±7.7% compared with day 1; *n*=5, *P*=0.323 versus day1, paired *t*-test; *P*=0.0316 versus Non-HAB, *t*-test). Scale bars: (**b**,**e**,**g**), 20%; *P<0.05; **P<0.01; ***P<0.0005; ****P<0.0001. Error bars±s.e.m.

Next, we assessed the impact of the environment on behaviours observed following photoactivation of PVN CRH neurons. We photostimulated PVN CRH neurons in a novel environment (Novel) and footshock chamber (following footshock) in CRH^eYFP^ and CRH^ChR2^ mice. Photostimulation increased grooming in the novel environment (Fig. 6a–c) and in the footshock chamber (Fig. 6d–f). We next tested whether the behavioural repertoire in response to optical stimulation is modulated by environmental familiarity by comparing the behaviour of mice in novel cages and cages to which they had been habituated. Optically evoked grooming time was gradually attenuated as the presumptive threat level of the context increased (Fig. 6g,h), but the distribution of grooming time was unchanged (Supplementary Fig. 11a). The reliable decrease in grooming behaviour from HC to novel to footshock chamber suggests that greater familiarity with the environment is likely a positive signal for grooming behaviour. To test this idea, we conducted experiments in two groups of mice. In the first group, mice were habituated by exposing them to the testing chamber on each of five successive days. In the second group, mice were not exposed to the chamber. Both groups were then introduced to the chamber and grooming was quantified in response to blue light delivery on each of five successive days (Fig. 6i). The HAB animals showed no change in total distance travelled (Fig. 6j and Supplementary Fig. 11b) or in grooming time (Fig. 6k and Supplementary Fig. 11c) in response to photostimulation. By contrast, in non-HAB animals, there was a decrease in total distance travelled (Fig. 6j and Supplementary Fig. 11b) and an increase in grooming (Fig. 6k and Supplementary Fig. 11c) on each of the five successive days. CRH^eYFP^ mice only showed insignificant levels of grooming in either condition (Supplementary Fig. 11d). These observations demonstrate that the perceived familiarity of the environment positively impacts PVN CRH-driven grooming behaviour.

### PVN CRH photoactivation blunts context appropriate behaviours

**Figure 7:**
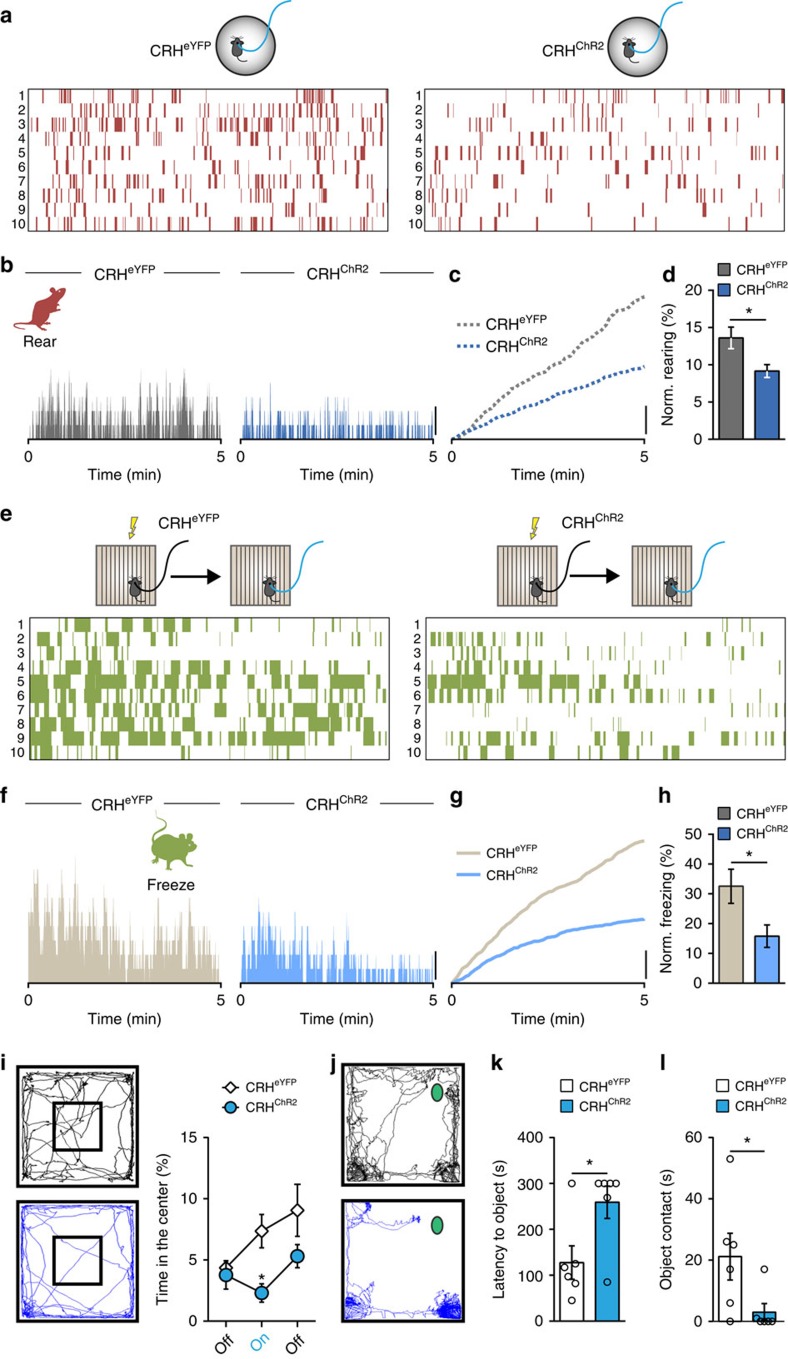
Photostimulation of PVN CRH^ChR2^ neurons overrides contextual cues. (**a**–**d**) Optical stimulation of PVN CRH neurons attenuates rearing in novel environment (Novel). (**a**) Each row represents an individual animal. (**b**) Histograms showing percentage of animals rearing. (**c**) Cumulative graphs demonstrate the relative extent of rearing. (**d**) Rearing time as a fraction of all behaviours after exclusion of time spent grooming (CRH^eYFP^: 13.6±1.4%, *n*=10; versus CRH^ChR2^: 9.2±0.9%, *n*=10; *P*=0.0165; *t*-test). (**e**–**h**) Optical stimulation of PVN CRH neurons disrupts freezing in FS. (**e**) Each row represents an individual animal. (**f**) Histograms show percentage of animals freezing. (**g**) Cumulative graphs demonstrate the relative extent of freezing. (**h**) Quantification of fractional freezing time if time spent grooming is excluded from the analysis (CRH^eYFP^: 32.5±5.7%, *n*=10; versus CRH^ChR2^: 15.8±3.8%, *n*=10; *P*=0.0251; *t*-test). (**i**) Assessment of locomotion in an open field test. Representative locomotor trajectory plots during optical stimulation in CRH^eYFP^ (black) and CRH^ChR2^ (blue) mice. Accompanying graph shows CRH^ChR2^ mice spend significantly less time spent in the centre zone during photostimulation (CRH^eYFP^: before: 3.9±0.5%, during: 6.1±0.5%. after: 6.6±0.9, *n*=16; versus CRH^ChR2^: before: 4.3±1.0%, during: 3.3±0.8%, after: 5.3±0.8%, *n*=14; CRH^eYFP^ during versus CRH^ChR2^ during, *P*=0.0291; repeated-measures two-way ANOVA). (**j**) Representative locomotor trajectory plots during optical stimulation in CRH^eYFP^ (black) and CRH^ChR2^ (blue) mice in a novel object (green shape) test. Optical stimulation reduces exploration of a novel object as measured by the latency to touch (**k**, CRH^eYFP^: 127.7±36.4 s, *n*=6; versus CRH^ChR2^: 259.0±35.2 s, *n*=6; *P*=0.0267; *t*-test) and the time spent in close proximity (**l**, CRH^eYFP^: 21.2±7.6 s, *n*=6; versus CRH^ChR2^: 3.0±2.8 s, *n*=6; *P*=0.0494; *t*-test). Scale bars: (**b**,**c**,**f**,**g**), 20%; *P<0.05; Error bars±s.e.m.

Finally, we asked whether the relationship between the environment and behaviours observed in response to stimulation of PVN CRH neurons was reciprocally modulated. In other words, could direct activation of PVN CRH neurons over-ride environmental cues? Here we compared the dominant behaviours in the novel and footshock environments in CRH^eYFP^ and CRH^ChR2^ animals in response to photostimulation. Photostimulation decreased rearing in the novel environment (Fig. 7a–d and Supplementary Fig. 12a–d) and freezing in the footshock chamber (Fig. 7e–h and Supplementary Fig. 12e–h). We then asked whether this environmental ‘over-ride' ability of CRH neurons was limited to the behaviours we have described, or whether this hints at a broader role of PVN CRH neurons. To test this idea, we assessed the effects of CRH photostimulation in two widely used behavioural tests, the open field and the novel object recognition. In the open field, the CRH^ChR2^ mice spent less time in the centre of the open field during the 5-min photostimulation period (Fig. 7i) but locomotion was unaffected (Supplementary Fig. 12i). Next, we asked whether activation of PVN CRH neurons would interfere with novel exploration behaviour (Fig. 7j). During the 5-min observation period, the natural exploration of a novel object was virtually eliminated when CRH neurons were photostimulated (Fig. 7k,l). These observations indicate that PVN CRH neurons decrease the sensitivity of mice to contextual cues. During this time, mice resort to self-directed grooming activity instead of exhibiting behavioural patterns that are consistent with engaging with their environment.

## Discussion

An acute stress necessitates an immediate behavioural and physiological response^1^,^2^. Here we combined cell-specific optogenetic targeting with the assessment of multiple behaviours to demonstrate that PVN CRH neurons orchestrate a complex repertoire of behaviours after an acute stress. This behavioural repertoire does not require endocrine signalling, but rather relies on an excitatory, glutamatergic projection to a subset of neurons in the perifornical region of the LH. Furthermore, although these behaviours are exquisitely sensitive to environmental context, the selective activation of CRH neurons can over-ride the environmental cues, resulting in behaviours that appear mismatched to the context. These findings provide a new framework for assessing behaviours after stress and suggest that animals de-escalate their behaviours after stress in a specific pattern that is influenced by the environment and the activity of PVN CRH neurons.

PVN CRH neurons are viewed as the canonical endocrine controllers of the stress response^12^, but they may also regulate complex behaviours following stress^16^. One of the most well-studied behaviours after stress is grooming^7^, and consistent with previous findings, we observed reliable increases in grooming after stress. Grooming, however, was one of eight distinct behaviours that we quantified. In naïve animals, these behaviours were spontaneous (that is, no external stimulus) with a clear bias towards surveying the HC environment. Following stress, there was an abrupt re-organization of the extant behaviours and a significant re-allocation of the time spent on specific behaviours. In addition to the increase in grooming, there were also increases in walking and rearing. These exploratory behaviours were evident early in the observation period, but waned within a few minutes, suggesting they may play a role in threat assessment following a stressful event^5^. Although all three behaviours were increased after stress, photoinhibition of CRH neurons selectively decreased grooming and increased rearing and walking. Meanwhile, photoactivation of PVN CRH neurons in the absence of stress did not mimic the behaviours observed after stress, but increased grooming and decreased rearing and walking. Collectively, these observations suggest that the firing of PVN CRH neurons, in the absence of immediate stress, may decrease behaviours associated with risk assessment in favour of behaviours that are self-directed. Consistent with this idea, recruitment of these cells even in environments that demand increased vigilance (novel environment, FS chamber) increased grooming, suggesting that PVN CRH neurons are important in matching appropriate behaviours to the environmental context.

Optical recruitment of CRH neurons increased circulating CORT, but *ex vivo* recordings show the axons of these cells release glutamate at synapses in the perifornical region of LH. Our double retrograde labelling showing that a subpopulation of cells in PVN projects to both targets is consistent with a previous report that neuroendocrine CRH neurons sending branching collaterals to adjacent hypothalamic regions^15^; it is unclear whether double-labelling of some, but not all, CRH neurons represents an under-sampling of the population because of technical limitations, or whether this suggests that information from a seemingly homogenous population of neurons can be routed to different targets depending on the information being conveyed^29^. Our findings that PVN CRH neurons control behaviour independent of their ability to release hormone, CRH is consistent with previous work demonstrating that CRF knockout mice have a normal grooming following stress^30^. This adds to the growing body of work demonstrating that putative collaterals from neuroendocrine cell populations in the hypothalamus can modulate behaviour^31^,^32^,^33^; importantly, we provide one of the first demonstrations that presumptive neuroendocrine cells not only modify, but also drive behaviour selection. This may be part of a larger theme suggesting that hypothalamic circuits participate in behaviours that extend beyond those that are strictly need based or homeostatic^34^. Indeed, it appears that the hypothalamus exerts a bottom up control of complex behaviours^35^,^36^,^37^ and that the CRH neurons play an essential role in shifting behavioural attention away from the environment and towards behaviours that are more internally focused.

Recent efforts to establish causal links between behaviour and underlying neural substrates have been extremely fruitful; here we further these efforts but with an important distinction. Rather than examining a single behaviour in isolation, we took an approach built on observations made by early behaviouralists who commented on individual behaviours as one component of a more complex pattern or ethogram of behaviours in the animal's natural environment^11^. In some aspects, the behavioural ethogram we describe is a broader representation of microstructure of individual behaviours that has recently been demonstrated^38^. These authors conclude that the individual elements of a given behaviour represent a library of physical motifs that can be re-purposed and re-sequenced to generate distinct behaviours. As we did not observe any ‘new' behaviours after stress, we would put forward a similar analogy here suggesting that individual behavioural traits are indeed re-purposed into more complex behavioural programmes. Our observations provide evidence for a distinct ethogram following stress that is controlled by PVN CRH neurons. This ethogram is exquisitely sensitive to environment, but this sensitivity is muted by activation of PVN CRH neurons.

New insights gained from approaches that establish causal links between innate behaviour and neural circuits are an important step in furthering our understanding of how the brain controls complex behaviour in a changing environment. They also set the stage for further explorations that use circuit-based approaches to better understand neurodevelopmental and psychiatric disorders^39^. By pinpointing an essential node in the brain for controlling behaviours immediately after an acute stress, our observations provide a new model that can be exploited to better understand the circuit function/dysfunction underlying stress disorders. For example, increased arousal and hypervigilance that persist after a traumatic event could be a consequence of rapid decreases in PVN CRH activity after stress. By contrast, the ability of PVN CRH neurons to dampen the impact of environmental cues suggests that increased activity in this cell population may contribute to stereotyped behaviours linked to psychiatric and neurodevelopmental disorders in which individuals eschew their environmental context and turn their focus towards self-directed or internally focused behaviours. Specifically, intense inward focus is a core feature in autism spectrum disorder, depression and anxiety. In each of these conditions, patients exhibit internally focused behaviours ranging from stereotypies to ruminations to negative thinking. Strikingly, repetitive stereotyped behaviours, particularly following exposure to stressful situations are a cardinal feature of autism spectrum disorder^10^. Recent animal models have directed attention towards the amygdala as a key structure that controls the balance between social and stereotyped behaviour^40^,^41^, but our findings would suggest an expanded model that incorporates PVN CRH neurons in regulating these behaviours, specifically after stress, is warranted.

## Methods

### Animals

All experiments were approved by the University of Calgary Animal Care and Use Committee in accordance with Canadian Council on Animal Care guidelines. *Crh-IRES-Cre* (*B6(Cg)-Crhtm1(cre)Zjh/J*; stock number 012704) and *Ai14* (*B6.Cg-Gt(ROSA)26Sortm14(CAG-TdTomato)Hze/J*; stock number 007914) mice, whose generation has been detailed previously^42^,^43^, were obtained from Jackson laboratories. Colony maintenance and genotyping was carried out as described previously^17^. For experiments, *Crh-IRES-Cre*+/+ mice and offspring derived from crosses of homozygous *Crh-IRES-Cre* and *Ai14* genotypes were used. Until the first procedure mice were group-housed, then single-housed on a 12:12 h light/dark schedule (lights on at 07:00 hours) with *ad libitum* access to food and water.

### Injection and implantation

In a stereotaxic apparatus under isoflurane anaesthesia, glass capillaries were lowered into the brain of 6- to 8-week-old *Crh-IRES-Cre*;*Ai14* mice (anteroposterior (AP), −0.7 mm; lateral (L), −0.3 mm from the bregma; dorsoventral (DV), −4.5 mm from the dura). Recombinant adeno-associated virus (AAV) carrying ChR2-eYFP (Addgene plasmid 20298, pAAV-EF1a-double floxed-hChR2(H134R)-eYFP-WPRE-HGHpA; 5 × 10^11^ GC per ml; Penn Vector Core), eYFP (Addgene plasmid 20296, pAAV-EF1a-double floxed-eYFP-WPRE-HGHpA; 5 × 10^11^ GC per ml; Penn Vector Core) or Arch3.0-eYFP (rAAV2-EF1a-double floxed-eArch3.0-eYFP; 5 × 10^11^ GC per ml; UNC Vector Core) was pressure injected with Nanoject II apparatus (Drummond Scientific Company) in a total volume of 210 nl. Mice were allowed to recover at least 14 days before further experiments. For *in vivo* optogenetic experiments, mono fiberoptic cannulas (Doric Lenses) were stereotactically implanted under similar conditions (for Arch3.0: AP, −0.7 mm; L, 0.0 mm from the bregma; DV, −4.0 mm from the dura, for ChR2 AP, −0.7 mm; L, −0.3 mm from the bregma; DV, −4.0 mm from the dura; for LH stimulation: AP, −1.2 mm L, −1.0 mm from the bregma; DV, −4.2 from the dura). Animals were allowed to recover for a week and handled ca 5 min daily for 4 consecutive days before all behavioural testing.

For neural tracing experiments, 6- to 8-week-old *Crh-IRES-Cre*;*Ai14* mice were pressure injected with green retrobeads (Lumafluor) as described above (AP, −1.2 mm; L, −1.0 mm from the bregma; DV, −5.2 mm from the dura) in a total volume of 32 nl. One week later, mice received 100 μl ml^−1^ of fluorogold (4 mg ml^-1^; Fluorochrome) injection i.v. Mice were killed and processed for immunohistochemistry after one additional week.

### Slice preparation and electrophysiology

Three to five weeks post AAV-injection, mice were anaesthetized via isoflurane inhalation and decapitated. Rapidly dissected brains were immersed in slicing solution (0–4 °C, 95% O_2_/5% CO_2_ saturated) containing (in mM): 87 NaCl, 2.5 KCl, 0.5 CaCl_2_, 7 MgCl_2_, 25 NaHCO_3_, 25 D-glucose, 1.25 NaH_2_PO_4_, 75 sucrose. A vibratome (Leica) was used to prepared coronal hypothalamic slices (250 μm thickness), which were transferred for 1+hours before recording in artificial cerebrospinal fluid (32 °C, 95% O_2_/5% CO_2_ saturated) containing (in mM): 126 NaCl, 2.5 KCl, 26 NaHCO_3_, 2.5 CaCl_2_, 1.5 MgCl_2_, 1.25 NaH_2_PO_4_, 10 glucose. Recordings were performed in artificial cerebrospinal fluid (1 ml min^−1^ perfusion) at 30–32 °C. The following drugs were applied via perfusion pump: DNQX (10 μM, Tocris), picrotoxin (100 μM, Sigma), 4-aminopyridine (4-AP, 500 μM, Tocris) and tetrodotoxin (TTX) (1 μM, Tocris). PVN/LH neurons were identified using differential interference contrast and epifluorescence optics (UVICO, Rapp Optoelectronics) and a camera (AxioCam MRm) on an upright microscope (Zeiss). Borosilicate electrodes (3–5 mΩ tip) were backfilled with recording solution composed of (in mM) 108 K-gluconate, 2 MgCl_2_, 8 Na-gluconate, 8 KCl, 1 K_2_-EGTA, 4 K_2_-ATP, 0.3 Na_3_-GTP, 10 mM HEPES, 10 mg ml^−1^ biocytin. Signals were amplified (Multiclamp 700B, Molecular Devices), low-pass filtered (1 kHz), digitized (10 kHz, Digidata 1322) and recorded (pClamp 9.2) for offline analysis. After recordings, slices were fixed in 4% paraformaldehyde (PFA; 24 h), incubated with streptavidin-A555 (1:500) and cleared in 50:50 glycerol/tris-buffered saline (TBS), before mounting and confocal imaging.

### Optogenetics

For *in vitro* recordings, a micromanipulator-attached fibre optic cable (105 μm core diameter) delivered light from a laser (for ChR2: 473 nm, OptoGeni 473, IkeCool Corporation; for Arch3.0: 593 nm, IKE-593-100-OP, IkeCool Corporation) was placed 1–2 mm away from the target area. Light intensity was calibrated by a Photodiode Power Sensor (Thorlabs). Maximally, 2.5 or 15 mW light (for ChR2 or Arch3.0, respectively) was delivered to the tissue.

For *in vivo* experiments, the light source (for ChR2: 473 nm, LRS-0473-GFM, Laserglow Technologies; for Arch3.0: 593 nm, IKE-593-100-OP, IkeCool Corporation) was connected to the implanted ferrule with a fibre optic cable (200 μm core diameter, Doric Lenses). The lasers were controlled with a manually programmable Master 8 unit (A.M.P.I.).

### Behavioural assessment

Wild-type mice received a series of footshocks (0.5 mA for 2 s, ten times in 5 min; SMSCK, Kinder Scientific) and their activity was video-recorded for 15 min in the footshock chamber, in a novel environment or in the HC immediately after the footshock. For *in vivo* optogenetics experiments, mice were handled the 4 days preceding behavioural assessment. Blue light was delivered for 5 min (10 Hz, 10 ms pulse width, 15 mW; 473 nm); in LH stimulation experiments, 20 Hz was used. Yellow light (15 mW) was delivered continuously for 15 or 5 min. Behavioural video analysis was conducted by an individual blinded to subject treatment group using a macro in Microsoft Excel. Walking was noted as the animal changed its location or turned as long as the front paws moved. Freezing behaviour was noted if animal showed no movement for at least 3 s, except respiratory movements.

For c-Fos immunolabelling, animals were killed 2 h after light stimulation. For experiments with Arch3.0, after recovery mice were handled for 4 days and HAB to the experimental condition for 3 additional days. After a similar series of footshock, their behaviour was recorded in their HC under continuous yellow light (15 mW) for 15 min. For assessing the involvement of circulating CORT, metyrapone (75 mg per kg, Tocris Bioscience, dissolved in 50 μl polyethylene glycol) was administered i.p. 60 min before footshock. For circulating CORT level measurements, baseline blood samples were taken from the tail vein at least 2 h before light stimulation. Second sampling was done 15 min after the onset of light stimulation. CORT level was measured using an ELISA kit (Arbor Assays). To investigate social cues bedding was not replaced in the HC of mice for 10 days prior testing. Experiment subjects were at the age of 16 weeks while conspecific males were 7 weeks old.

### Immunohistochemistry

To prepare fixed brain tissue, mice were anaesthetized with sodium pentobarbital (30 mg kg^−1^) and transcardially perfused with phosphate-buffered saline, followed by 4% PFA in phosphate buffer (4 °C). Brains were placed in PFA 24 h followed by 20% sucrose phosphate buffer. 30 μM coronal brain sections were obtained via cryostat in three series. Rinses were performed before/between incubations with TBS containing triton (TBSt; pH 7.4, with 0.1% Triton X-100), blocking solution (5% normal donkey serum in TBSt) was pre-applied for 1 h and used in subsequent antibody incubations. Rabbit anti-c-Fos Ab5 (1:10,000 dilution; overnight at room temperature; Calbiochem) primary antibody or rabbit anti-fluorogold (1:10,000, overnight at room temperature; Chemicon) was used. For fluorogold and c-Fos labelling, biotinylated donkey anti-rabbit secondary antibody (1:500; Jackson ImmunoResearch) and DyLight-405-conjugated streptavidin were used (1:500; Jackson ImmunoResearch) in *Crh-IRES-Cre;Ai14* animals, whereas in *Crh-IRES-Cre* animals, Alexa-555-conjugated donkey anti-rabbit (1:500; Molecular Probes) was utilized. Slide-mounted and coverslipped sections were imaged using a confocal microscope (Olympus BX50 Fluoview and Nikon D-Eclipse C1). For c-Fos assessment, we included the entire rostral-caudal extent of one side of the PVN and the region around the fornix. Immunolabelled nuclei were counted using ImageJ.

### Analysis and statistics

Where quantification was made, data are represented as mean±standard error of the mean (s.e.m.). Statistical analysis was performed in GraphPad Prism 6 using paired and unpaired Student's *t*-test to for two group comparisons, whereas repeated measures one-way and two-way ANOVA with Bonferroni's multiple comparison *post-hoc* test for sequential treatment data. *P* values less than 0.05 were considered significant.

### Data availability

The data that support the findings of this study are available from the corresponding author upon request.

## Additional information

**How to cite this article:** Füzesi, T. *et al.* Hypothalamic CRH neurons orchestrate complex behaviours after stress. *Nat. Commun.* 7:11937 doi: 10.1038/ncomms11937 (2016).

## Supplementary Material

Supplementary Information Supplementary Figures 1-12

Supplementary Movie 1 Behavior of CRHeYFP control mice after stress

Supplementary Movie 2 Photoinhibition of PVN CRH neurons disrupts behavioral repertoire after stress

Supplementary Movie 3 Photostimulation of PVN CRH Neurons initiate grooming

Supplementary Movie 4 Behavior in novel environment after stress exposure

Supplementary Movie 5 Behavior in unsafe environment after stress exposure
