# Supplementary material for: Hypothalamic CRH neurons orchestrate complex behaviours after stress
Source: Nat Commun. 2016 Jun 16;7:11937. doi: 10.1038/ncomms11937 (PMC4912635; doi:10.1038/ncomms11937)
Supplement: Supplementary Information — Supplementary Figures 1-12 [file ncomms11937-s1.pdf]

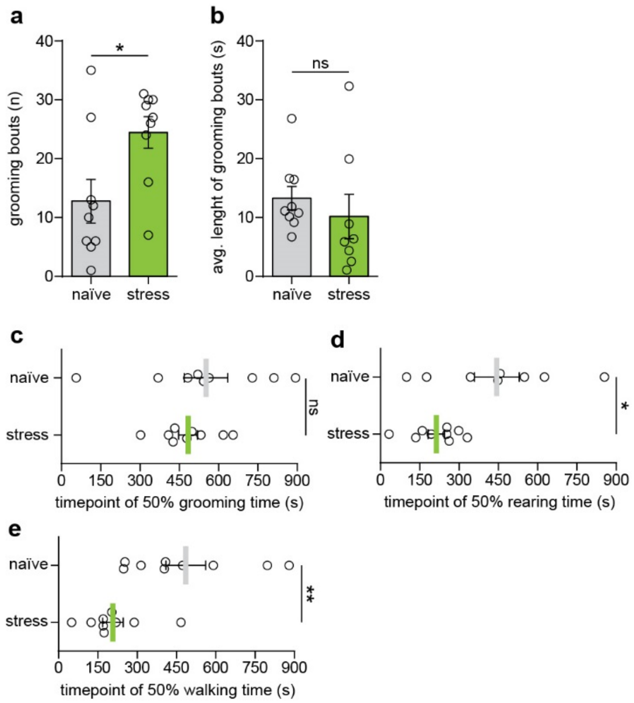

Supplementary Figure 1. Quantification of grooming and median timepoints of behaviors
a, Quantification of grooming bouts of naïve and footshocked mice immediately following stress (naïve: 12.8 ± 3.7, n=9; stressed: 24.4 ± 2.7, n=9; p=0.0214; t-test). **b**, Average length of grooming bouts is similar (naïve: 10.2 ± 3.8, n=9; stressed: 13.3 ± 2.0, n=9; p=0.4625; t-test). **c**, Quantification of the median timepoint of grooming behavior (naïve: 552.1 ± 83.4, n=9, p=0.2558 vs HT, one sample t-test; stressed: 483.6 ± 36.2, n=9, p=0.3812 vs HT, one sample t-test; p=0.4622 vs naïve; t-test). **d**, Quantification of the median timepoint of rearing behavior (naïve: 444.5 ± 86.0, n=8, p=0.9507 vs HT, one-sample t-test; stressed: 213.4 ± 31.0, n=9, p<0.0001 vs HT, one-sample t-test; p=0.0182 vs naïve; t-test). **e**, Quantification of the median timepoint of walking behavior (naïve: 485.6 ± 76.0, n=9, p=0.6523 vs HT, one-sample t-test; stressed: 207.5 ± 39.1, n=9, p=0.0003 vs HT, one-sample t-test; p=0.0050 vs naïve; t-test). HT: halftime of observation period; ns: not significant; *p<0.05; **p<0.01; Error bars, ± s.e.m.

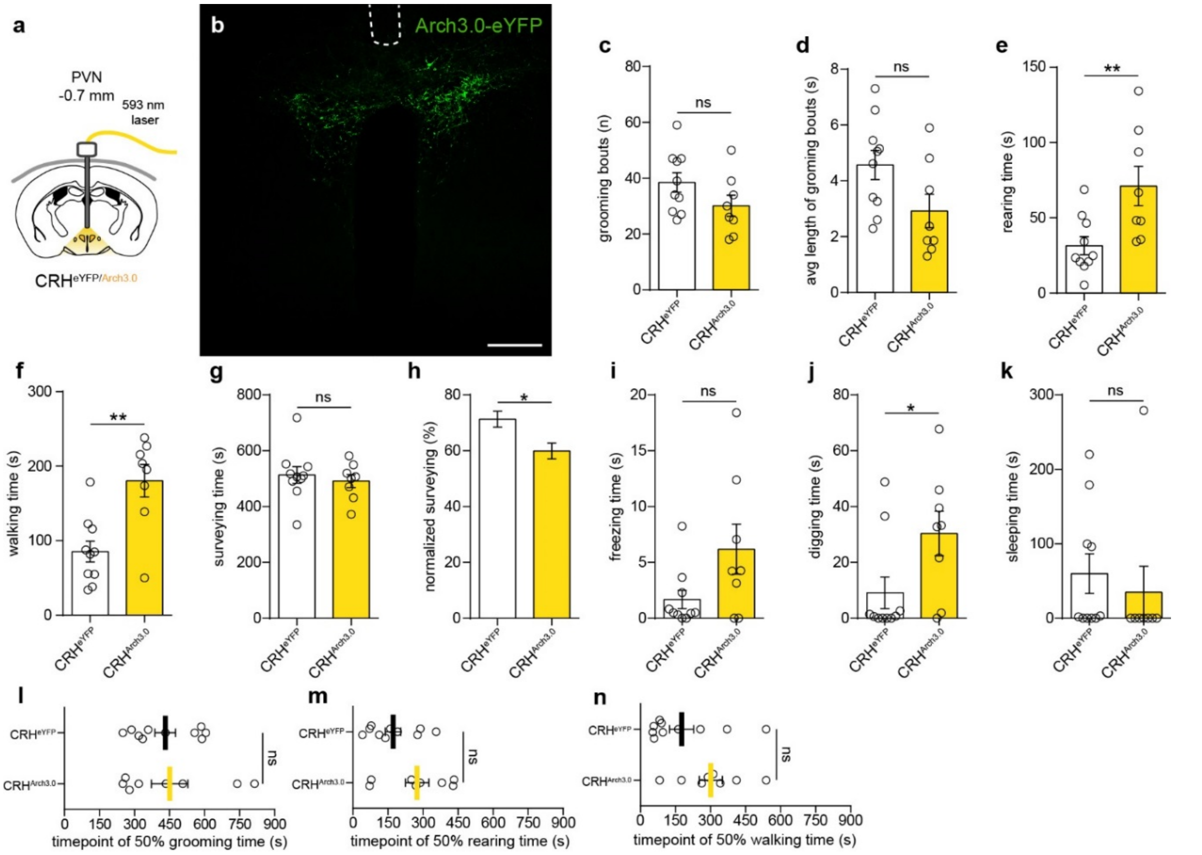

Supplementary Figure 2. Effects of *in vivo* photoinhibition of CRH$^{Arch3.0}$ neurons on behavior
Schematic map (**a**) and low magnification image (**b**) shows the implantation site of the light ferrule (dashed line) for experiments utilizing optical inhibition of PVN CRH neurons. **c**, Quantification of grooming bouts during optical silencing of PVN CRH neurons (CRH^{eYFP}: 38.5 ± 3.5, n=10 vs CRH$^{Arch3.0}$: 30.1 ± 3.8, n=8; p=0.1247; t-test). **d**, Average length of grooming bouts (CRH^{eYFP}: 4.6 ± 0.5, n=10 vs CRH$^{Arch3.0}$: 2.9 ± 0.6, n=8; p=0.0542; t-test). **e**, Rearing is increased during photoinhibition (CRH^{eYFP}: 31.4 ± 6.0 n=10 vs CRH$^{Arch3.0}$: 71.1 ± 13.1, n=8; p=0.0093; t-test). **f**, Walking time also increases during optical inhibition of PVN CRH neurons (CRH^{eYFP}: 85.4 ± 14.0, n=10 vs CRH$^{Arch3.0}$: 180.4 ± 21.7, n=8; p=0.0015; t-test). **g**, Total time spent surveying during optical silencing of PVN CRH neurons is unchanged (CRH^{eYFP}: 513.1 ± 29.8, n=10 vs CRH$^{Arch3.0}$: 491.7 ± 23.4, n=8; p=0.5966; t-test). **h**, Fractional surveying time, if time spent grooming is excluded from the analysis is decreased (CRH^{eYFP}: 71.3 ± 2.9 %, n=10; vs CRH$^{Arch3.0}$: 59.9 ± 2.8 %, n=8; p=0.0132; t-test). Quantification of time spent freezing (**i**, CRH^{eYFP}: 1.7 ± 0.8 s, n=10; vs CRH$^{Arch3.0}$: 6.2 ± 2.2 s, n=8; p=0.0567; t-test), digging (**j**, CRH^{eYFP}: 9.1 ± 5.7 s, n=10; vs CRH$^{Arch3.0}$: 30.3 ± 8.0 s, n=8; p=0.0405; t-test) and sleeping (**k**, CRH^{eYFP}: 60.0 ± 26.6 s, n=10; vs CRH$^{Arch3.0}$: 34.9 ± 34.9 s, n=8; p=0.5680; t-test) during optical inhibition. **l**, Quantification of the median timepoint of grooming behavior (CRH^{eYFP}: 432.0 ± 44.2 s, n=10; vs CRH$^{Arch3.0}$: 450.6 ± 78.2, n=8; p=0.8308; t-test). **m**, Quantification of the median timepoint of rearing behavior (CRH^{eYFP}: 171.6 ± 33.3 s, n=10; vs CRH$^{Arch3.0}$: 275.0 ± 50.1, n=8; p=0.0944; t-test). **n**, Quantification of the median timepoint of walking behavior (CRH^{eYFP}: 176.4 ± 51.8 s, n=10; vs CRH$^{Arch3.0}$: 301.4 ± 49.2, n=8; p=0.1053; t-test). Scale bar: **b**, 200 µm; ns: not significant; *p<0.05; Error bars, ± s.e.m.

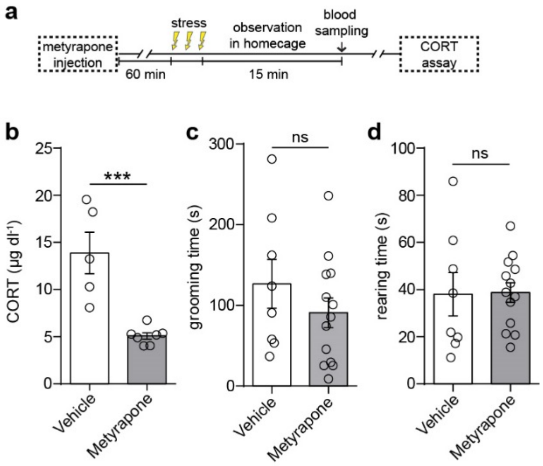

Supplementary Figure 3. CORT is unnecessary for the behaviors emerging after stress
a, Schematic experimental model. **b**, Quantification of the effect of metyrapone injection on stress induced CORT (vehicle: 13.9 ± 2.2 µg dl^{-1}, n=5; metyrapone: 5.1 ± 3.5 µg dl^{-1}, n=7; p=0.0009; t-test). **c**, Grooming time following footshock is unaffected by metyrapone (vehicle: 126.7 ± 30.3 s, n=8, vs metyrapone: 90.8 ± 18.4 s, n=13; p=0.29; t-test). **d**, Similarly, metyrapone has no effect on rearing behavior after stress (vehicle: 38.0 ± 9.1 s, n=8; metyrapone: 38.8 ± 4.2 s, n=13; p=0.9338; t-test). ns: not significant; ***p<0.0005; Error bars, ± s.e.m.

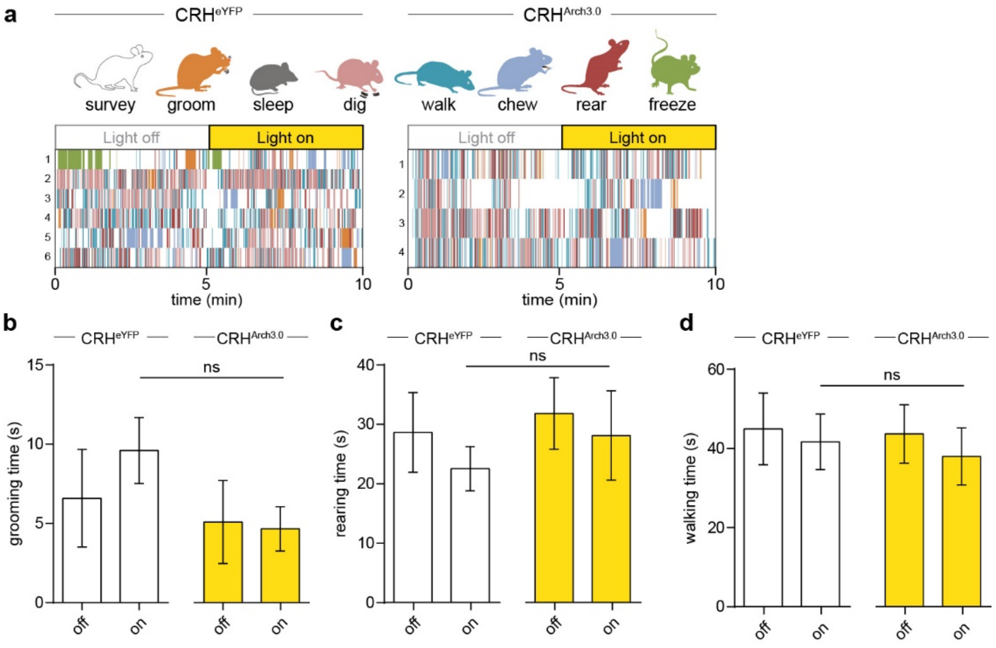

Supplementary Figure 4. ***In vivo*** **photoinhibition of CRH$^{Arch3.0}$ neurons in the absence of stress**
a, Detailed analysis shows 8 different behaviors observed in CRH^{eYFP} (left) and CRH$^{Arch3.0}$ (right) animals in the absence of stress for 5 min before and 5 min during photoinhibition. Each row represents one animal. **b**, Bar graphs show the quantification of grooming in CRH^{eYFP} (left) and CRH$^{Arch3.0}$ (right) mice before (off) and during (on) yellow light delivery (CRH^{eYFP} on: 9.6 ± 2.1 s, n=6; CRH$^{Arch3.0}$ on: 4.7 ± 1.4 s, n=4; p=0.1181; t-test). **c**, Quantification of rearing behavior (CRH^{eYFP} on: 22.6 ± 3.7 s, n=6; CRH$^{Arch3.0}$ on: 28.1 ± 7.5 s, n=4; p=0.4807; t-test). **d**, Quantification of walking behavior (CRH^{eYFP} on: 41.7 ± 7.0 s, n=6; CRH$^{Arch3.0}$ on: 38.0 ± 7.2 s, n=4; p=0.7319; t-test). ns: not significant; Error bars, ± s.e.m.

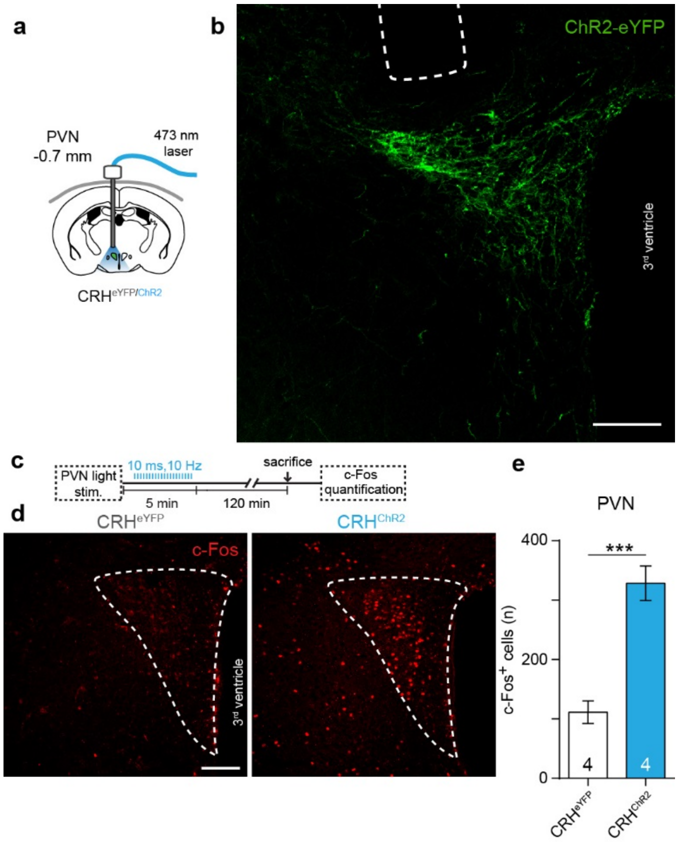

Supplementary Figure 5. *In vivo* photostimulation of PVN CRH^{ChR2} neurons

Schematic map (**a**) and low magnification image (**b**) shows the implantation site of the light ferrule (dashed line) for experiments utilizing optical activation of PVN CRH cell bodies. **c**, Optical stimulation resulted in a robust increase in the number of c-Fos positive cells in the PVN (dashed line) of CRH^{ChR2} animals 2 hours after the activation of CRH neurons (**d**). **e**, Bar graph shows the summary data of c-Fos analysis in PVN (CRH^{eYFP}: 95.0 ± 11.7, n=4; vs CRH^{ChR2}: 328.5 ± 29.1, n=4; p=0.0003; t-test). Scale bar: **b** and **cii**, 100 µm; ***p<0.0005; Error bars, ± s.e.m.

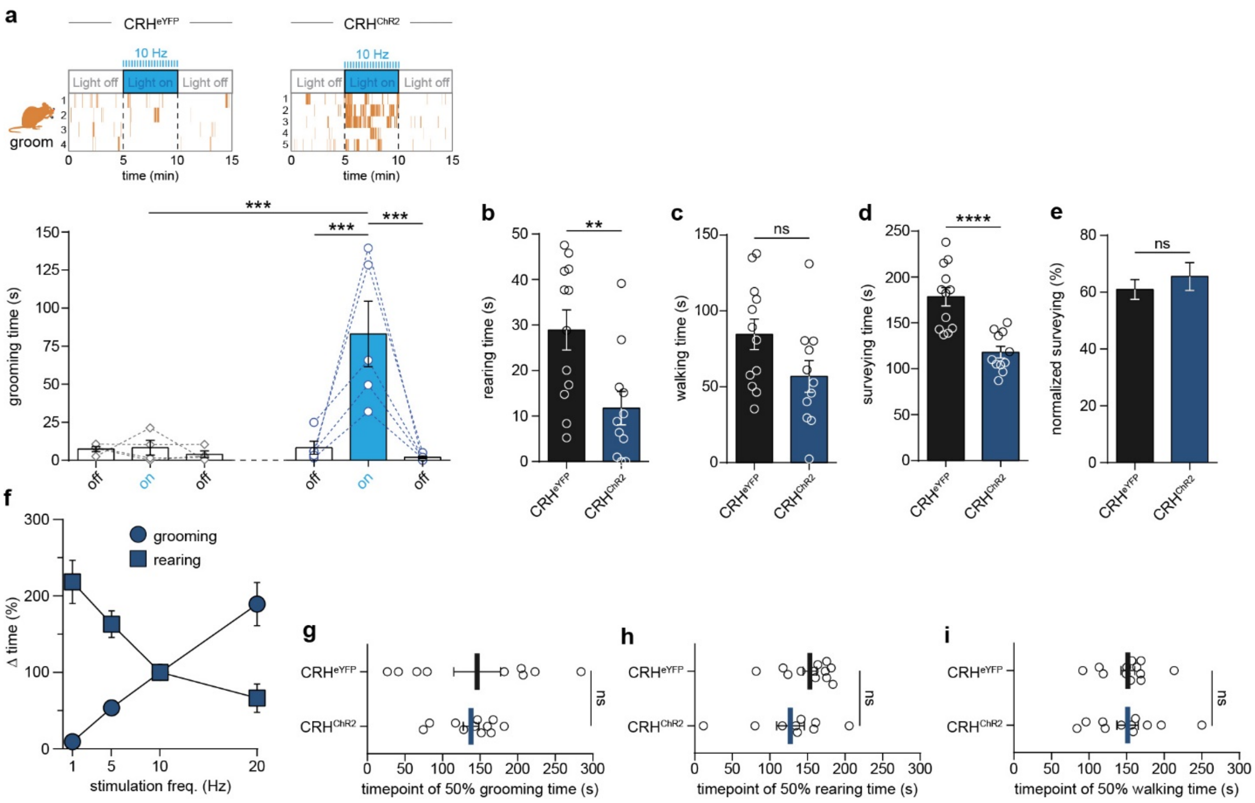

Supplementary Figure 6. Behavioral quantification of PVN CRH^{ChR2} photostimulation
a, Top, Detailed analysis of grooming behavior observed in CRH^{eYFP} (left) and CRH^{ChR2} (right) mice before, during and after of 5 min of optical stimulation in an observational chamber to which they were previously habituated in the absence of stress. Each row represents one animal. Bottom, Quantification of grooming in 5 min blocks before, during, and after photostimulation in CRH^{eYFP} (grooming time during light stimulation: 8.3 ± 4.9 s; n=4) and CRH^{ChR2} mice (grooming time during light stimulation: 83.1 ± 21.5 s, n=5; p=0.0001 vs CRH^{eYFP}; repeated measures 2-way ANOVA). Increase of grooming is limited to the stimulation period (CRH^{ChR2} before: 8.4 ± 4.2 s, p=0.0003 vs during; CRH^{ChR2} after: 2.1 ± 0.8 s, p=0.0001 vs during, p>0.9999 vs before; repeated measures 2-way ANOVA). **b**, Rearing is significantly inhibited (CRH^{eYFP}: 28.9 ± 4.4 s, n=12; vs CRH^{ChR2}: 11.7 ± 3.7 s, n=11; p=0.0073; t-test). **c**, Walking time is not significantly affected by optical stimulation (CRH^{eYFP}: 84.6 ± 10.0 s, n=12; vs CRH^{ChR2}: 56.9 ± 10.4 s, n=11; p=0.0695; t-test). **d**, Surveying time decreases during photostimulation (CRH^{eYFP}: 178.7 ± 10.0 s, n=12; vs CRH^{ChR2}: 118.0 ± 6.4 s, n=11; p<0.0001; t-test). **e**, However fractional surveying time as a fraction of all non-grooming behaviors is unaltered (CRH^{eYFP}: 61.0 ± 3.5 %, n=12; vs CRH^{ChR2}: 65.5 ± 4.9 %, n=11; p=0.4514; t-test). **f**, Increasing frequency of photostimulation in PVN increased total grooming time (circles) and decreases rearing time (squares). Photostimulation of PVN CRH neurons does not shift the median timepoint of grooming (**g**, CRH^{eYFP}: 146.1 ± 31.1 s, n=9; vs CRH^{ChR2}: 137.9 ± 10.4, n=11; p=0.7910; t-test), rearing (**h**, CRH^{eYFP}: 153.2 ± 9.0 s, n=12; vs CRH^{ChR2}: 127.3 ± 18.4, n=9; p=0.1877; t-test) or walking behavior (**i**, CRH^{eYFP}: 151.6 ± 9.1 s, n=12; vs CRH^{ChR2}: 151.2 ± 14.2, n=11; p=0.9809; t-test). ns: not significant; **p<0.01; ***p<0.0005; ****p<0.0001; Error bars, ± s.e.m.

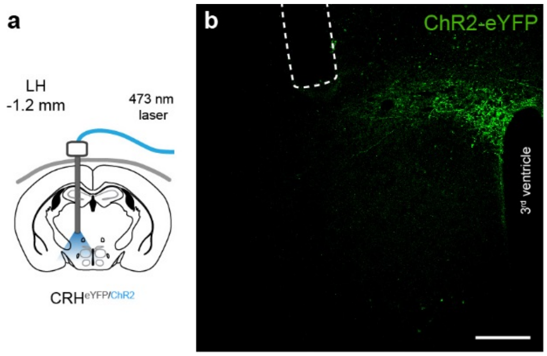

Supplementary Figure 7. Fiber placement for photostimulation in the LH
Schematic map (**a**) and low magnification image (**b**) of the unilateral light ferrule placement (dashed line) in ChR2-eYFP (green) injected mice. Scale bar: **b**, 100 µm

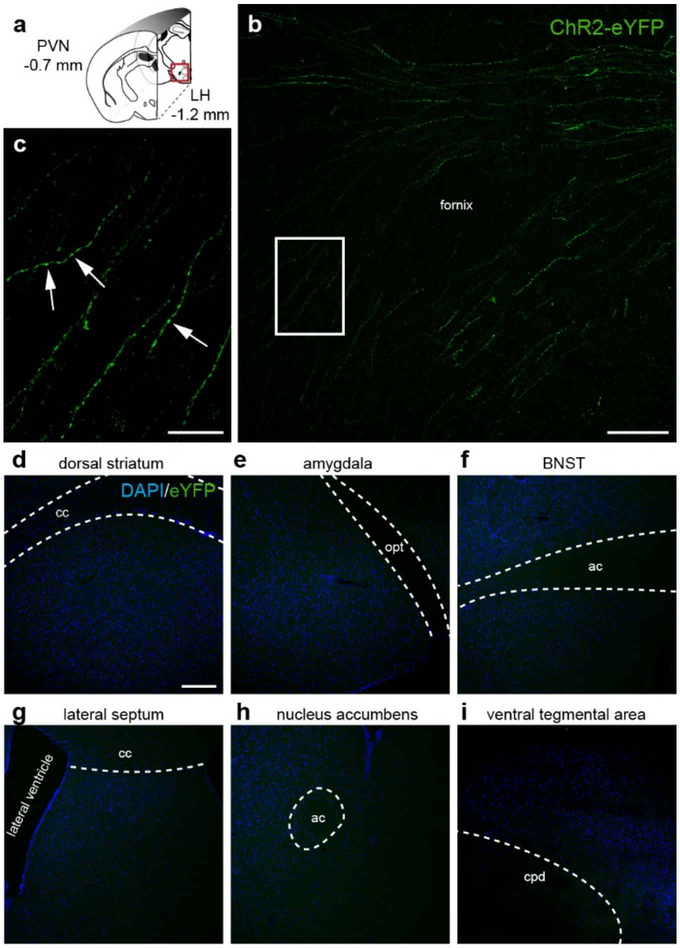

Supplementary Figure 8. PVN CRH fibers mapping reveals no projection targets outside the lateral hypothalamus

a, Schematic map (**a**) and low magnification image (**b**) show fibers originating from PVN CRH neurons labelled by ChR2-YFP expression in the lateral hypothalamus (LH). Note the bouton shaped structures (arrows) on ChR2-eYFP fibers in the high magnification image (**c**). Low magnification images demonstrate the lack of CRH fibers arising from the PVN in regions implicated in the mediation of stress response or grooming, such as the dorsal striatum (**d**), amygdala (**e**), bed nucleus of stria terminalis (BNST, **f**), lateral septum (**g**), nucleus accumbens (**h**) and the ventral tegmental area (**i**). Dashed lines represent the boundaries of various brain structures. cc: corpus callosum; opt: optic tract; ac: anterior commissure; cpd: cerebral peduncle. Scale bars: **b**, 50 μm; **c**, 25 μm; **d**-**i**, 200 μm.

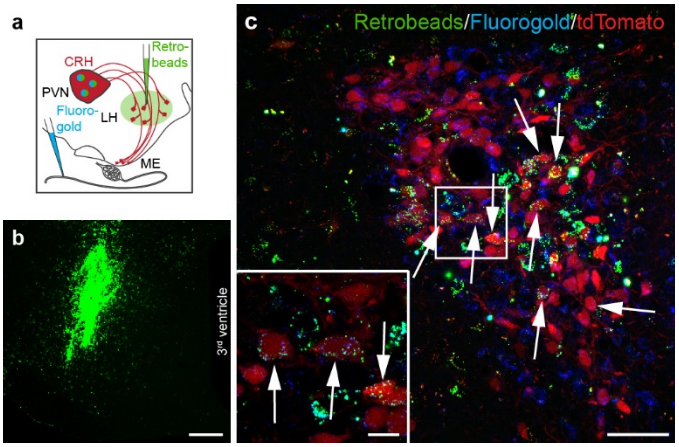

Supplementary Figure 9. A subset of PVN CRH neurons send collaterals to the median eminence and to the LH

a, Combined administration of Retrobeads into the LH and Fluorogold injection i.v. was used to identify the projection target of PVN CRH neurons. **b**, Low magnification image shows the Retrobeads injection site. **c**, Fluorogold (blue) and Retrobeads (green) co-localize in a subset of PVN CRH neurons (red) demonstrating that individual neurons simultaneously project to the pituitary and the LH. Inset, High magnification image of the labelled region, arrows point to triple-labelled neurons. Scale bars: **b**, 300 µm; **c**, 50 µm; **c** inset, 10 µm

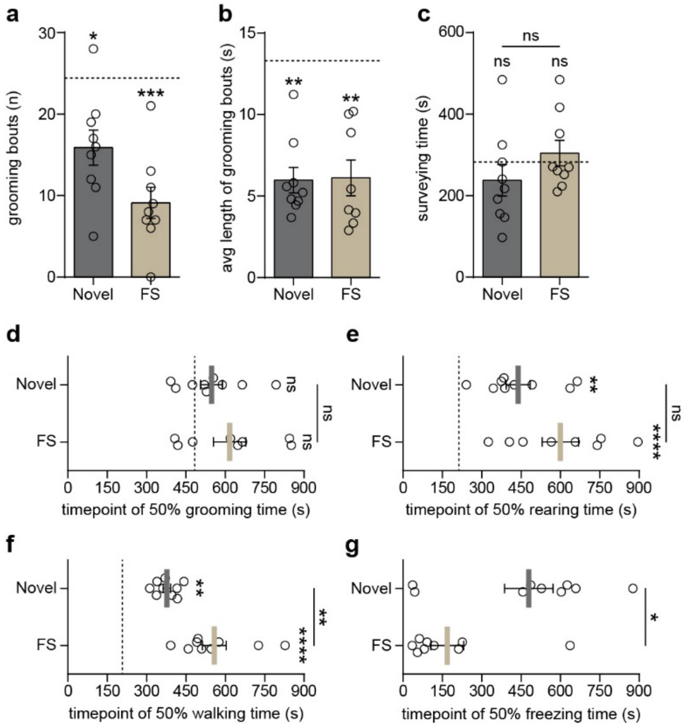

Supplementary Figure 10. Behavioral analysis of context sensitive behavioral repertoires following stress

Behavioral analysis of mice immediately after footshock in novel environment (Novel) and in the footshock cage (FS). **a**, Quantification of grooming bouts (dotted line represents mean grooming bouts in homecage (HC); Novel: 15.9 ± 2.2 s; FS: 9.1 ± 1.9 s; Novel vs HC p=0.0399; FS vs HC p=0.0002; n=9 in each group; 1-way ANOVA). **b**, Quantification of the average length of a grooming bout (dotted line represents mean grooming bout length in HC; Novel: 6.0 ± 0.8 s; FS: 6.1 ± 1.1 s; Novel vs HC p=0.0033; FS vs HC p=0.0051; n=9 in HC and Novel, n=8 in FS; 1-way ANOVA). **c**, Quantification of surveying (dotted line represents mean surveying time in HC; Novel: 237.9 ± 38.7 s; FS: 304.5 ± 31.1 s; Novel vs HC p>0.9999; FS vs HC p>0.9999; Novel vs FS p=0.5353; n=9 in each group; 1-way ANOVA). **d**, Quantification of the median timepoint of grooming behavior (dotted line represents median grooming time in HC; Novel: 547.8 ± 41.7 s, n=9; FS: 616.6 ± 61.7 s, n=8; p=0.1593; 1-way ANOVA). **e**, Quantification of the median timepoint of rearing behavior (dotted line represents median rearing time in HC; Novel: 438.9 ± 45.8 s, n=9; FS: 600.0 ± 69.3 s, n=8; Novel vs HC p=0.0098; FS vs HC p<0.0001; Novel vs FS p=0.0977; 1-way ANOVA). **f**, Quantification of the median timepoint of walking behavior (dotted line represents median walking time in HC; Novel: 377.1 ± 14.5 s; FS: 558.2 ± 45.6 s; Novel vs HC p=0.0078; FS vs HC p<0.0001; Novel vs FS p=0.0044; n=9 in each group; 1-way ANOVA). **g**, Quantification of the median timepoint of freezing behavior (Novel: 479.6 ± 92.4 s, n=9; FS: 169.0 ± 62.6 s, n=9; Novel vs FS p=0.0133; t-test). ns: not significant; *p<0.05; **p<0.01; ***p<0.0005; ****p<0.0001; Error bars, ± s.e.m.

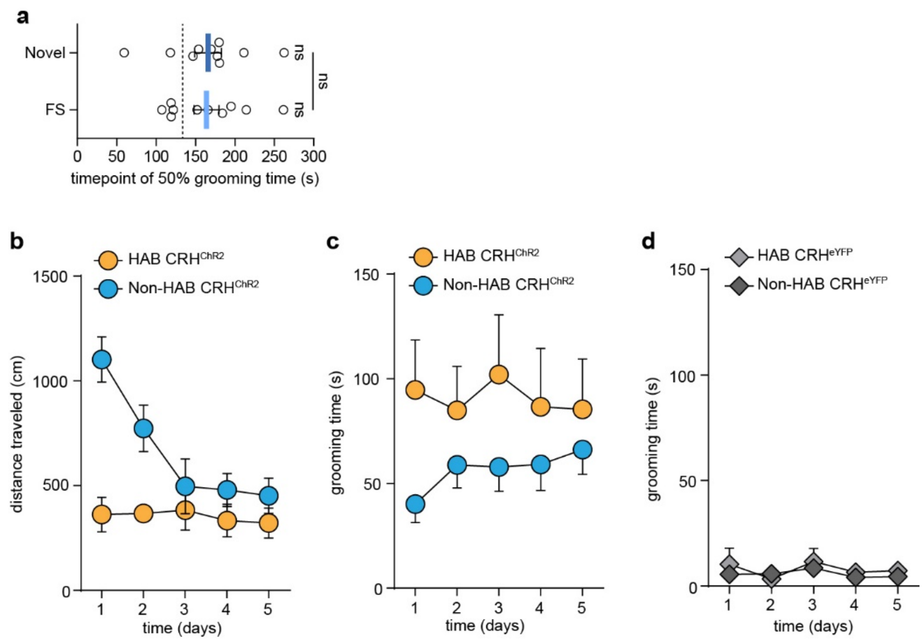

Supplementary Figure 11. Impact of context familiarity on photostimulation induced grooming

a, The temporal distribution of photostimulated grooming is similar in each contexts (dotted line represents median grooming time in habituated environment (HAB); Novel: 166.0 ± 17.0 s; FS: 164.0 ± 15.9 s, n=10; p=0.1947, repeated measures 1-way ANOVA). **b**-**d**, Graded effect of repeated photostimulation in CRH^{ChR2} mice. **b**, Bar graph of shows the gradual change of baseline locomotion distance of non-habituated (Non-HAB) and habituated (HAB) animals during the 5-day repeated exposure to the open field. **c**, Gradual change of grooming time during optical stimulation is demonstrated in non-HAB and HAB mice. **d**, CRH^{eYFP} mice did not show notable grooming response to light delivery on any day. ns: not significant; Error bars, ± s.e.m.

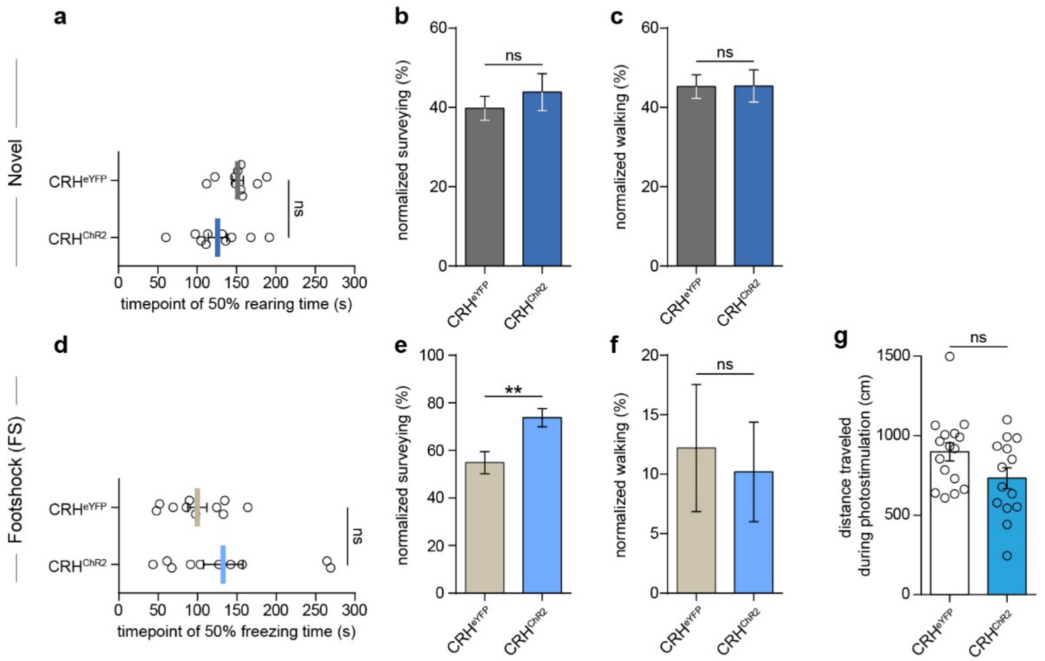

Supplementary Figure 12. Behavioral assessment of photostimulation overriding contextual cues

a, Quantification of the median timepoint of rearing behavior in novel environment (Novel) during photostimulation (CRH^{eYFP} : 151.6 ± 7.1 s, n=10; CRH^{ChR2}: 126.0 ± 11.8, n=10; p=0.0779; t-test). **b**, Quantification of rearing during optical stimulation in Novel (CRH^{eYFP}: 39.6 ± 4.2 s, n=10; vs CRH^{ChR2}: 20.1 ± 2.8 s, n=10; p=0.0012; t-test). **c**, Surveying time as a fraction of all non-grooming behaviors is unaffected by photostimulation in Novel (CRH^{eYFP}: 39.8 ± 3.0 %, n=10; vs CRH^{ChR2}: 43.9 ± 4.7 %, n=10; p=0.4723; t-test). **d**, Walking time as a fraction of all non-grooming behaviors is also unaltered by photostimulation in Novel (CRH^{eYFP}: 45.3 ± 3.0 %, n=10; vs CRH^{ChR2}: 45.4 ± 4.0 %, n=10; p=0.9756; t-test). **e**, Quantification of the median timepoint of freezing behavior after footshock in the footshock cage (FS) during photostimulation (CRH^{eYFP} : 99.7 ± 12.1 s, n=10; CRH^{ChR2}: 132.6 ± 25.1, n=10; p=0.2532; t-test). **f**, Quantification of freezing during optical stimulation in FS (CRH^{eYFP}: 96.2 ± 17.1 s, n=10; vs CRH^{ChR2}: 43.7 ± 11.0 s, n=10; p=0.0190; t-test). **g**, Surveying time as a fraction of all non-grooming behaviors in FS is elevated during photostimulation (CRH^{eYFP}: 54.9 ± 4.6 %, n=10; vs CRH^{ChR2}: 73.8 ± 3.8 %, n=10; p=0.0055; t-test). **h**, Walking time as a fraction of all non-grooming behaviors is unchanged by photostimulation in FS (CRH^{eYFP}: 12.2 ± 5.3 %, n=10; vs CRH^{ChR2}: 10.2 ± 4.2 %, n=10; p=0.7692; t-test). **i**, Locomotion is not significantly different during photostimulation in open field (CRH^{eYFP}: 898 ± 57 cm, n=16; vs CRH^{ChR2}: 732 ± 66 cm, n=14; p=0.0651; t-test). ns: non-significant; **p<0.01; Error bars, ± s.e.m.
